# Hemispheric segregation is evident in early perception, not just higher cognition

**DOI:** 10.3389/fpsyg.2026.1879495

**Published:** 2026-09-17

**Authors:** Petunia Reinke, Sebastian Ocklenburg

**Affiliations:** 1 Department of Psychology, Medical School Hamburg (MSH), Hamburg, Germany; 2 Institute for Cognitive and Affective Neuroscience (ICAN), Medical School Hamburg (MSH), Hamburg, Germany; 3 Biopsychology, Institute of Cognitive Neuroscience, Faculty of Psychology, Ruhr University Bochum, Bochum, Germany

**Keywords:** lateralization, EEG, handedness, hemispheric asymmetries, hemispheric segregation, N1

## Abstract

**Introduction:**

Functional hemispheric segregation, the organization of distinct cognitive functions into separate hemispheres, has so far been investigated mainly using behavioral measures or fMRI. Whether such segregation manifests at early perceptual stages, however, remains unknown. The present study is the first to test Gerrits' segregation bias model using EEG, examining lateralization of early ERP components (N1/N170) across language, face, and emotional face processing.

**Methods:**

The present study is based on an openly available EEG dataset obtained from OpenNeuro. We analyzed data from left-, mixed-, and right-handed participants to investigate the influence of handedness and sex on early functional hemispheric segregation. Hemispheric lateralization was assessed using early event-related potential components (N1/N170) across language, face, and emotional face processing tasks.

**Results:**

Consistent with predictions, typical lateralization patterns were most frequent, yet substantial individual variability was observed. Notably, “crowding” of multiple functions within one hemisphere – either left or right – was among the most common patterns, highlighting the probabilistic nature of hemispheric organization. These effects emerged at early perceptual stages, indicating that hemispheric segregation is not solely a consequence of later cognitive or decisional processes.

**Discussion:**

We suggest that differences in individual processing styles may partly explain variability in lateralization, particularly for simple recognition tasks. Our findings extend the segregation bias model by demonstrating that functional hemispheric segregation is already detectable during early perceptual processing and is modulated by both handedness and sex. This study underscores the importance of EEG for capturing the temporal dynamics of hemispheric organization and provides a novel perspective on the interplay between individual differences and early neural specialization.

## 1 Introduction

The human brain, like many other biological systems, is asymmetrically organized (Toga and Thompson, 2003). The differences between the left and right hemisphere manifest on macro-, micro-, and molecular scales, as well as in their functional specialization. This functional specialization is, for example, easily observable in everyday life: most people (around 90%) strongly favor their right hand for fine motor activities, while only one in ten uses their left hand (Papadatou-Pastou et al., 2020). Next to handedness, footedness and eye-dominance have also been found to show remarkable asymmetries on the population level, with approximately 76.3% showing a right-foot dominance (Packheiser et al., 2020) and 80.1% a right-eye dominance (Marcori et al., 2026).

Importantly, as described above, these asymmetries are not restricted to the individual level (e.g., one person favoring the right hand and the next the left hand) but show consistent population-level biases, where most individuals in a population show asymmetries in the same direction. Beyond motor and sensory domains, hemispheric asymmetries are also a fundamental characteristic of cognitive functions. As early as Broca (1861) presented compelling evidence for a leftward lateralization of language. Since then, hemispheric asymmetries have been found for almost all cognitive functions (Ocklenburg and Güntürkün, 2024). Despite the broad evidence for hemispheric asymmetries across cognitive domains, considerable heterogeneity exists with respect to how such asymmetries are assessed. While some studies focus on behavioral measures, such as dichotic listening tasks (Kimura, 1967; Hugdahl and Westerhausen, 2016; Westerhausen, 2019), chimeric faces tasks (Gupta and Pandey, 2010), hand preference, hand performance, or foot preference (Marcori et al., 2026), other studies use neuroimaging to assess which hemisphere is most involved in a specific task. Functional magnetic imaging (fMRI), for example, revealed a right hemispheric dominance for face processing (Frässle et al., 2016), and visual-spatial tasks (Hugdahl et al., 2006), as well as a left hemispheric dominance for language processes (Hirnstein et al., 2013). Findings regarding emotional processing are more mixed; however also suggest that the right hemisphere plays a key role in processing emotions (Beraha et al., 2012; Wyczesany et al., 2018). Complementing hemodynamic imaging approaches, such as fMRI, electroencephalography (EEG) provides a temporally highly resolved measure of neural activity. This allows the investigation of hemispheric asymmetries at very early stages of stimulus processing, reflecting initial perceptual and attentional processes rather than later, higher-order cognitive stages. One such early event-related potential (ERP), known as the N1 or the N170 depending on the research tradition, has been shown to be sensitive to lateralized processing of facial (Reinke et al., 2024), emotional (Zhang et al., 2011), and language-related stimuli (Spironelli and Angrilli, 2007; Grossi et al., 2010; Proverbio, 2022). The label N170 is most commonly used for the face-sensitive component, whereas N1 is the more general term also applied to word- and language-related responses. Although these responses may not share identical neural generators across tasks, they occur within the same early time window and index early perceptual lateralization in each case. We therefore use the combined term N1/N170 when referring to them collectively, and the task-specific labels (N170 for faces and emotional faces, N1 for words) where this aids clarity. In line with findings from fMRI, here also a right hemispheric dominance for face (Reinke et al., 2024) and emotion processing (Prete et al., 2018) was found, as well as a left hemispheric dominance for language processing (Spironelli and Angrilli, 2007; Emmorey et al., 2017; Bisiacchi and Cainelli, 2022; Proverbio, 2022). While the N1/N170 has been widely used as a marker of hemispheric lateralization, evidence on the lateralization of the earlier P1 component remains comparatively sparse. For example for face processing, a recent review found consistent lateralization effects for the N170, but not for the earlier P1 component (Reinke et al., 2024).

The large population-level asymmetry of most cognitive functions leads to some functions being more often located within the same hemisphere (e.g., emotion and face processing), while other functions usually reside in different hemispheres (e.g., language and face processing). These patterns are described as functional integration (co-localization) and functional segregation (separation of functions). Whereas lateralization describes the predominant hemispheric involvement of a single function, functional hemispheric segregation refers to the co-localization or separation of multiple functions across the two hemispheres (Gerrits, 2024). Building on this distinction, Gerrits (2024) proposed a theoretical framework that postulates that every person has an innate segregation bias which can either be “typical” (typical functional segregation; TFS) or “reverse-typical” (reverse typical functional segregation, rTFS). It is proposed that around 99% of the population shows the TFS-bias, where each function tends to be lateralized to the typical hemisphere. Only 1% of the population is thought to show an rTFS-bias, where each function tends to lateralize to the opposite hemisphere. However, since it is thought, that each function lateralizes independently from other functions, also TFS individuals might occasionally show atypical patterns of segregation, and rTFS individuals might show more typical patterns. However, in both groups, more extreme deviations from the innate bias (e.g., reverse lateralization in TFS individuals and typical lateralization in rTFS individuals) occur progressively rarer. Importantly, earlier evidence suggests that disruptions of stereotypical segregation patterns may be associated with reduced cognitive performance in otherwise healthy individuals (Quin-Conroy et al., 2024), highlighting the potential functional significance of hemispheric organization.

Individual differences in functional hemispheric segregation, as described by Gerrits (2024), may be influenced by biological factors such as handedness and sex. For example, left-handed individuals more often exhibit atypical language lateralization to the right hemisphere (Willems et al., 2014), which may lead to co-localization of functions that are usually segregated, or separation of functions that are usually integrated. Similarly, sex-related differences have been reported for various cognitive domains, including language (Kansaku and Kitazawa, 2001), emotion (Wager et al., 2003; Bourne and Maxwell, 2010), and face processing (Proverbio et al., 2006; Godard and Fiori, 2010; Stasch et al., 2018). Even though there is some debate over the existence of sex differences in lateralization (Boles, 2005; Ortigue et al., 2005), studies that report sex differences often find that females tend to be less or even reverse lateralized compared to males (Voyer, 1996; Stasch et al., 2018; Proverbio, 2021). This suggests that biological factors may modulate patterns of lateralization and, therefore, functional hemispheric segregation. Whether and how these differences are reflected in early neural signatures of segregation, however, remains largely unclear.

Although Gerrits' framework does not directly specify when in the processing stream functional hemispheric segregation should manifest, it implies that individual differences in segregation patterns can be identified across tasks and functions. So far, empirical tests of this framework have relied primarily on behavioral measures (Marcori et al., 2026) and fMRI (Gerrits et al., 2020), which either lack direct neural indices or provide limited temporal resolution. Consequently, it remains unclear whether functional hemispheric segregation reflects early perceptual asymmetries or emerges at later stages of cognitive processing. Building on this, early ERP components such as the N1 and N170 provide a promising way to operationalize and test predictions about functional hemispheric segregation at early stages of cognitive processing. While many ERP studies focus on hemispheric differences for single cognitive functions, fewer studies have addressed how multiple functions are jointly organized across hemispheres. Especially early ERP asymmetries provide a suitable framework to investigate functional hemispheric segregation, as they allow the comparison of lateralization patterns across different tasks and stimulus domains within the same temporal window. For example, language and face-related processes can be examined with respect to whether they elicit lateralized N1/N170 responses in the same or in opposite hemispheres. Whether the emotional content of a face additionally influences this early asymmetry is not well established. While emotional expressions have been shown to modulate the N170 (Hinojosa et al., 2015), it remains unclear whether they also affect the direction of its lateralization, and competing theories of emotional lateralization make different predictions in this regard. Including both neutral and emotional faces therefore allows us to examine whether lateralization at this early stage is comparable for both, or whether emotional content shifts the asymmetry.

Taken together, early ERP asymmetries such as the N1 and N170 provide a promising approach to investigate functional hemispheric segregation at initial stages of cognitive processing. The present study therefore aims to examine functional hemispheric segregation in the EEG, focusing on early lateralized ERP components and the influence of handedness and sex on such segregation patterns. Based on the above-mentioned model of hemispheric segregation we hypothesize that:

1. During the words task, there will be a left hemispheric population level asymmetry of the N1 component.
2. During the faces and emotional faces task, there will be a right hemispheric population level asymmetry of the N170 component.
3. Typical functional segregation (TFS; i.e., left-lateralized N1 for words and right-lateralized N170 for faces/emotional faces) will occur significantly more frequently across participants than any other single lateralization pattern.
4. Lateralization patterns involving a reversal in only one domain (i.e., either word, face, or emotional face lateralization) will occur significantly more frequently across participants than patterns involving a reversal in two domains.
5. Reversed typical functional segregation (rTFS; i.e., right-lateralized N1 for words and left-lateralized N170 for faces/emotional faces) will occur significantly less frequently across participants than any other lateralization pattern.
6. Right-handed individuals will show a significantly higher frequency of TFS than left- or mixed-handed individuals.
7. Given evidence suggesting reduced or reversed hemispheric lateralization in females compared to males, females will show a significantly lower frequency of TFS (i.e., a significantly higher frequency of atypical lateralization patterns, defined as any pattern other than TFS) than males.

## 2 Methods

### 2.1 Dataset

The analysis was conducted using an openly available dataset from OpenNeuro (OpenNeuro Dataset ds007172). The dataset was produced and uploaded by our group and a full description of all tasks can be found in Reinke et al. (2026). The preprocessed data, as well as the data on handedness can be found in the OSF: https://osf.io/g74vq?view_only=cc40b6db1a9d4745b70164d417fbe731). The original study was approved by the ethics committee of the MSH Medical School Hamburg and was performed with the ethical standards laid down in the 1964 Declaration of Helsinki. All participants gave informed consent after being informed about the study. While the dataset was analyzed in a previous publication on the association between EEG ERP and oscillation asymmetries (Reinke et al., 2026), the current study is distinctly different on both the thematical and the analytical level.

### 2.2 Participants

The dataset comprised 100 native German speakers between 18–39 years. Of these, one had to be excluded due to bad data quality and one was excluded due to being of diverse gender. Even though it would be important to study this group of individuals in future studies, a sample size of one would not allow for meaningful statistics. The final sample therefore included 98 participants (53 females and 45 males), of which 40 were right-handed (18 females and 22 males), 31 were mixed-handed (21 females and 10 males), and 27 were left-handed (14 females and 13 males). Handedness was assessed using the Edinburgh Handedness Inventory (EHI; Oldfield, 1971) and the cut-offs were chosen based on a recent paper by Mundorf et al. (2024), who showed that cut-offs of −60 and +60 are optimal for investigating mixed-handedness. Participants with scores above 60 are classified as being right-handed, between – 60 and + 60 as mixed-handed, and below −60 as left-handed. All participants reported normal or corrected to normal vision, had no uni- or bilateral sensory or motor deficits, had no history of mental or neurological illnesses, and did not take medication. The original study was approved by the ethics committee of the MSH Medical School Hamburg and was performed with the ethical standards laid down in the 1964 Declaration of Helsinki.

### 2.3 EEG tasks

The given dataset contained data of three EEG tasks: a words task, a faces task, and an emotional faces task. A detailed description of all tasks and preprocessing steps can be found in Reinke et al. (2026). During the words task a short German word (mono- or bi-syllabic) was shown in the center of the screen and the participants were instructed to react to every animal name (frequency of 20%) by pressing the space button. There were two blocks, one where the participants had to answer with their right hand and one, where they had to use the left hand to answer. The faces and emotional faces tasks were constructed in the same way and participants had to react to all female faces (frequency of 20%). The faces task showed only neutral faces, while during the emotional faces task 80 happy, 40 angry, and 40 fearful expressions were shown to the participants. Each task comprised 160 stimulus presentations, and only participants with more than 100 valid epochs per task were included in the analysis. Task compliance was high, with a median of two errors (misses and false alarms) in the words task and one in the faces and emotional faces tasks. The tasks were easy and had the purpose to keep the participants engaged, as the main goal of the task was to induce and measure early perceptual asymmetries (measured as the N1/N170 ERP component) between both hemispheres.

### 2.4 Data preparation

For the current analysis we used the already preprocessed ERP data published on the OSF. Exact information on all preprocessing steps and topographical distributions of ERP components can be found in Reinke et al. (2026), the aspects relevant to the present analysis are summarized here. The EEG had been recorded from 32 gel-based active Ag/AgCl electrodes (actiCHamp, Brain Products GmbH, Gilching, Germany) arranged according to the international 10–20 system, sampled at 500 Hz and online-referenced to an electrode between Fz and Cz. During preprocessing with the MNE-BIDS-Pipeline and MNE-Python (Gramfort et al., 2013; Larson et al., 2026), the data had been re-referenced to the common average, high-pass filtered at 1 Hz, low-pass filtered at 40 Hz, and notch-filtered at 50 Hz. The continuous data had been segmented into epochs from −300 to 800 ms relative to stimulus onset, with the pre-stimulus interval serving as baseline. Ocular and other artifacts had been removed using independent component analysis, and remaining bad epochs had been rejected using an automatic peak-to-peak procedure. Topographic distributions and ERP waveforms for each task, confirming the parieto-occipital N1/N170, are provided in Reinke et al. (2026). For the analysis of typical and atypical patterns of lateralization we focused on posterior electrodes (O1/O2, P3/P4, and P7/P8). These sites are located over posterior visual areas and are among those commonly used to quantify the N1/N170 for both face and word stimuli (Gao et al., 2009; Fajkowska et al., 2011; Amora et al., 2022; Civera et al., 2024). Crucially, they are also the posterior sites at which lateralized N1/N170 effects were observed in the dataset's original study (Reinke et al., 2026). As hemispheric asymmetry is a prerequisite for functional segregation, we restricted our analysis to sites at which asymmetries had been established. Because these neighboring sites largely reflect the same posterior N1/N170 asymmetry, and because single electrodes are susceptible to noise, we averaged across them to obtain a more stable estimate of hemispheric dominance. For the faces and emotional faces task the peak-to-peak N170 amplitude was chosen, while for the words task, the mean amplitude was used. This was done because of a peculiarity of the used dataset, where very large P1 amplitudes tended to distort the amplitude measurements (for more information see Reinke et al., 2026). Analyses using a uniform measure across all tasks are provided in Supplementary material B, illustrating how the choice of measure affects the recovered direction of lateralization. The peak-to-peak amplitude was quantified as the difference between the P1 (90–130 ms) and the subsequent N1/N170 peak (130–220 ms) over posterior electrodes, while the mean amplitude was measured in the 130–220 ms window. Before determining the direction of lateralization, the mean of the amplitudes was calculated for left (O1, P3, P7) and right (O2, P4, P8) electrodes separately. After that, the direction of lateralization was determined in the way, that if the amplitude of right electrodes was more negative than amplitude of the left electrodes, participants were classified as having a rightward lateralization. If the amplitude of left electrodes was more negative, they were classified as being left dominant. We deliberately deviated from the continuous Asymmetry Index (AI; (Right – Left)/ (Right + Left)), which is a common approach in the field. The AI is only bounded within its interpretable range from −1 to 1 when the amplitudes of both hemispheres share the same sign. In the words task, however, the unusually large P1 amplitudes of the present dataset frequently kept the N1 within positive voltage values over one hemisphere, while the other hemisphere showed the expected negative deflection. When the two hemispheres carry opposite signs, the denominator (Right + Left) approaches zero and the AI becomes unstable, producing values far outside the −1 to 1 range (occasionally exceeding 100 in our data). Directly comparing which hemisphere is more negative is robust to this instability: it yields a valid direction of lateralization irrespective of the absolute sign of either hemisphere. For each task, a participant's lateralization was classified as typical or atypical relative to the expected direction: leftward lateralization was considered typical for the words task and atypical for the faces and emotional faces tasks, and vice versa. Furthermore, for each participant, the combination of the three task-specific lateralization directions (words, faces, and emotional faces) defined an individual segregation pattern, which was then classified as described in the following section. This way of quantifying segregation, as the combination of the categorical lateralization directions of the individual functions, corresponds to how segregation phenotypes are defined in the segregation bias model (Gerrits, 2024), and allows our data to be related to its predictions directly.

### 2.5 Analysis

Although prior literature suggests that hemispheric asymmetries are present at the population level, we wanted to explicitly test this assumption in our own dataset before proceeding to the classification of participants into typical and atypical segregation patterns. To this end, we first tested whether the proportion of participants showing typical lateralization differed significantly from chance level (50%), separately for each task and within each combination of sex (male/female) and handedness (left/mixed/right). A proportion significantly different from chance would indicate a systematic, population-level asymmetry. For this purpose, binomial tests were conducted separately for each group and task. The resulting *p*-values were adjusted for multiple comparisons across groups and tasks using the Holm method to control the family-wise error rate. Furthermore, chi-square tests were conducted for each task separately to investigate, whether the proportion of typical/atypical lateralization differed between all six groups.

To investigate which patterns of hemispheric segregation are present in the current dataset, we investigated how often each possible combination of lateralization patterns was present across all participants. The possible patterns are: 1) typical (Language-Left, Faces-Right, Emotional Faces-Right), 2) all reversed (Language-Right, Faces-Left, Emotional Faces-Left), 3) language reversed (Language-Right, Faces-Right, Emotional Faces-Right), 4) faces reversed (Language-Left, Faces-Left, Emotional Faces-Right), 5) emotions reversed (Language-Left, Faces-Right, Emotional Faces-Left), 6) faces and language reversed (Language-Right, Faces-Left, Emotional Faces-Right), 7) emotions and language reversed (Language-Right, Faces-Right, Emotional Faces-Left), 8) emotions and faces reversed (Language-Left, Faces-Left, Emotional Faces-Left). To analyze whether left- and right-handed participants show different patterns of hemispheric segregation, we first split the participants in two handedness groups based on the EHI, whereby participants with scores above zero were counted as right-handers and participants with scores of zero or below as left-handers. To facilitate interpretation the possible patterns were aggregated to: typical (TFS), all Reversed (rTFS), one reversed (1rFS; pattern 3, 4, and 5), and two reversed (2rFS; pattern 6, 7, and 8). The same procedure was applied to three handedness groups, where the cut-offs were chosen to be −60 and +60 (as described above) to group participants into left-, mixed-, and right-handers. Because previous results on this dataset suggest sex differences in lateralization patterns, we also analyzed males and females separately. Therefore, we investigated the lateralization patterns of six groups (left-, mixed-, and right-handed females and males).

To test, whether left-handers show more often a typical lateralization patterns compared to right-handers, we calculated chi-square tests of independence for each of the groupings listed above (between-group analyses). In addition, within each group we conducted pairwise chi-square comparisons between the segregation patterns to test which patterns differed significantly in frequency - in particular, whether TFS occurred more frequently than each of the other patterns (within-group analyses). All analyses were performed in R (R Core Team, 2023), using the *chisq.test()* function. Because several expected cell counts were below five, *p*-values were estimated using a Monte Carlo simulation with 10,000 permutations. For the pairwise within-group comparisons, *p*-values were adjusted for multiple comparisons using the Holm method. For each omnibus chi-square test, we report Cramér's V as a measure of effect size together with its 95% confidence interval. Cramér's V was bias-corrected (Bergsma, 2013), which is particularly appropriate here because the uncorrected statistic is upwardly biased in small samples; confidence intervals were derived analytically from the noncentral chi-square distribution. Because *p*-values were estimated via Monte Carlo simulation while confidence intervals for Cramér's V were derived from the noncentral chi-square distribution, a significant test result may co-occur with a confidence interval that includes zero, particularly for small subgroups; both quantities are reported as computed. Whenever necessary, we calculated standardized residual measurements (SRM) to see in which cells the count was significantly different from the expected values, with values being larger than |2| being considered as significant (Haberman, 1973).

### 2.6 Exploratory analysis

Given the unusually large P1 amplitudes in the present dataset, we applied the same lateralization approach described above to the P1 (analyzed as the mean amplitude across all tasks). Because the P1 is a positive component, the direction of lateralization was determined by which hemisphere showed the more positive amplitude. As we had no prior hypothesis about the direction of P1 asymmetry, we did not classify patterns as “typical” or “atypical”. Given the exploratory nature of these analyses, we focused on describing the observed P1 patterns rather than on extensive statistical testing, calculating chi-square tests only where appropriate. Results are reported in Supplementary material A.

## 3 Results

### 3.1 Patterns of hemispheric asymmetry across tasks

To assess if most participants show the expected rightward lateralization during the faces and emotional faces task (H2) and expected leftward lateralization during the words task (H1), we investigated the distribution of typical and atypical lateralization for each task separately (see Figure 1). As expected, the typical lateralization was overall the most common pattern, however with large differences between the groups.

**Figure 1:**
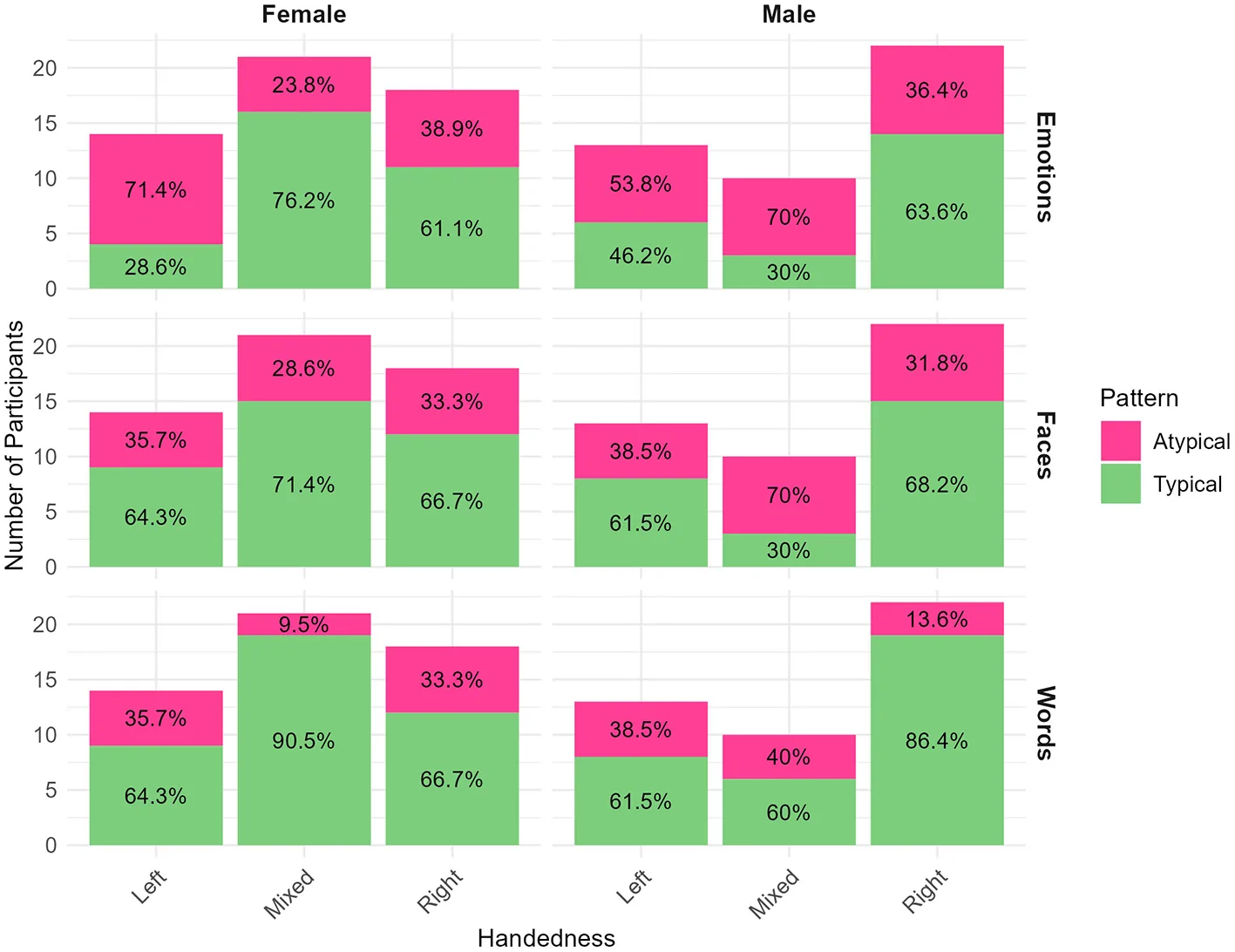
Hemispheric functional lateralization of left-, mixed-, and right-handed females **(left)** and males **(right)**. For the emotional faces **(top)** and faces **(middle)** tasks the typical lateralization pattern is a rightward N1 lateralization, while for the words task **(bottom)** the typical lateralization is thought to be leftward.

The chi-square tests did not show differences in lateralization between all groups for any of the tasks [emotional faces: *X*^2^= 11.64, *p*_*adj*_ = 0.120, *V* = 0.26, 95% CI (0.00, 0.44); faces: *X*^2^ = 5.70, *p*_*adj*_ = 0.341, *V* = 0.08, 95% CI (0.00, 0.29); words: *X*^2^=8.06, *p*_*adj*_ = 0.300, *V* = 0.18, 95% CI (0.00, 0.36)]. However, residual analysis suggested that during the emotional faces task, left-handed females showed a lower-than-expected proportion of typical lateralization (28.6%; SRM = −2.16) and mixed-handed females showed a higher-than-expected proportion of typical lateralization (76.2%; SRM = 2.19). For the faces task, only mixed-handed males showed a lower-than-expected proportion of typical lateralization (30.0%; SRM = −2.30). For the words task, no differences between the groups were identified.

To investigate whether the typical lateralization is more common than atypical lateralization, binomial tests were conducted for each group. After correction for multiple comparisons, only mixed-handed females (*p*_*adj*_ = 0.004) and right-handed males (*p*_*adj*_ =0.015) showed a significantly higher than chance proportion of typical lateralization during the words task (compared to atypical lateralization). No other group showed such a pattern during any of the tasks, suggesting that typical and atypical lateralization do not differ within each task and group.

### 3.2 Patterns of hemispheric segregation

Across all participants, different patterns of hemispheric segregation emerged. Figure 2 shows the distribution of the individual patterns. TFS was the most common pattern with 36.7% of participants showing this pattern. 26.5% of participants showed a pattern where faces and emotional faces showed a reverse lateralization, leading to all three functions being co-localized in the left hemisphere. Another 14.3% of participants show a pattern of reversed language lateralization, leading to all three functions being crowded in the right hemisphere. Normal language and face lateralization, with reversed emotional faces lateralization was shown by 9.2% of participants. RTFS was displayed by 6.1% of all participants. A pattern where language and faces are lateralized to the right and emotional faces are lateralized to the left was shown by 3.1% of participants. 2.0% of participants showed an atypical face lateralization to the left together with an atypical language lateralization to the right. Another 2.0% of participants showed an atypical left lateralization for faces, while emotional faces and language were typically lateralized to the right and left, respectively.

**Figure 2:**
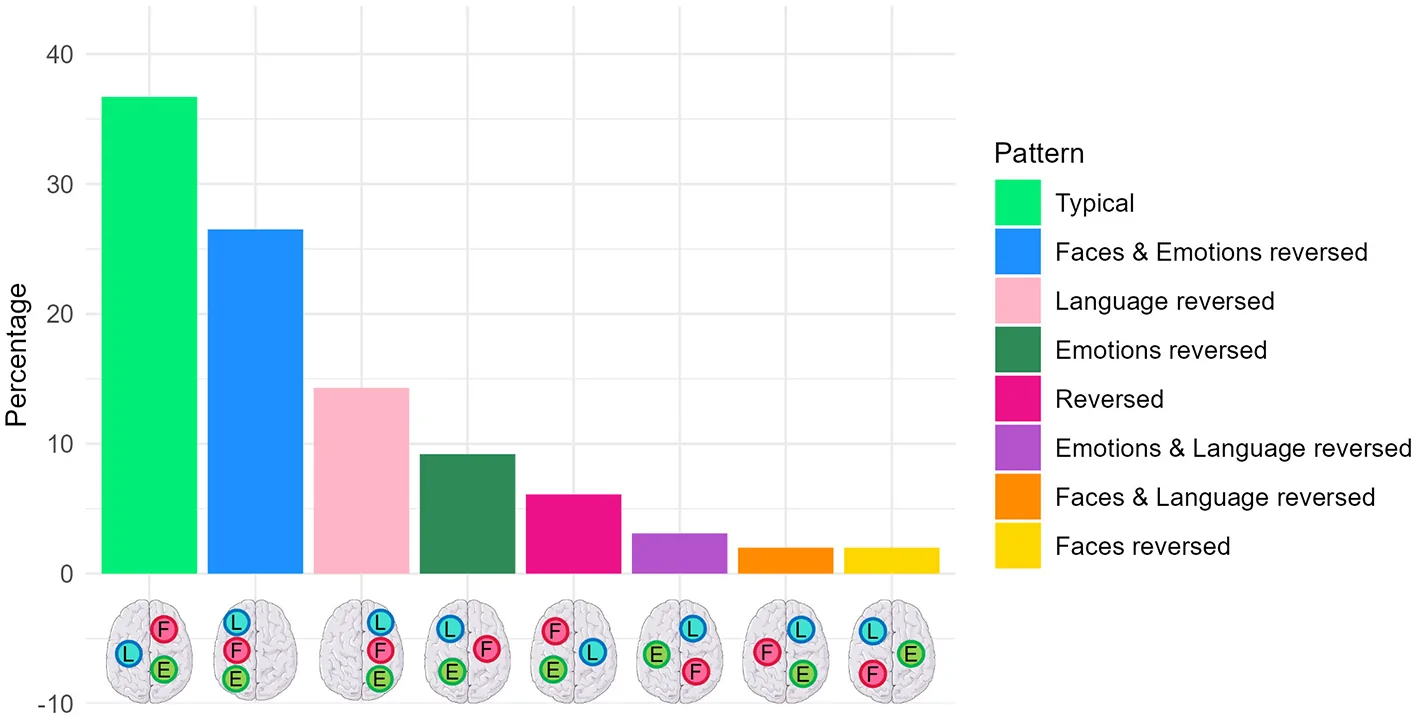
Patterns of phenotyping regarding hemispheric segregation within the whole sample. “F” denotes the faces task, “L” the language task, and “E” the emotional faces task.

### 3.3 Hemispheric segregation by handedness (left and right)

Dividing the sample into left- (EHI < 0) and right- (EHI >0) handers did not show the expected higher proportion of TFS in right-handers [*X*^2^ = 4.87, *p* = 0.191, *V* = 0.14, 95% CI (0.00, 0.35)]. However, on a descriptive level (see Figure 3), there seems to be a difference between both groups: while 42.6% of the right-handers showed an TFS pattern, only 29.5% of the left-handers showed TFS. This, however, is not reflected in the residual analysis, where none of the values were above our threshold of |2|.

**Figure 3:**
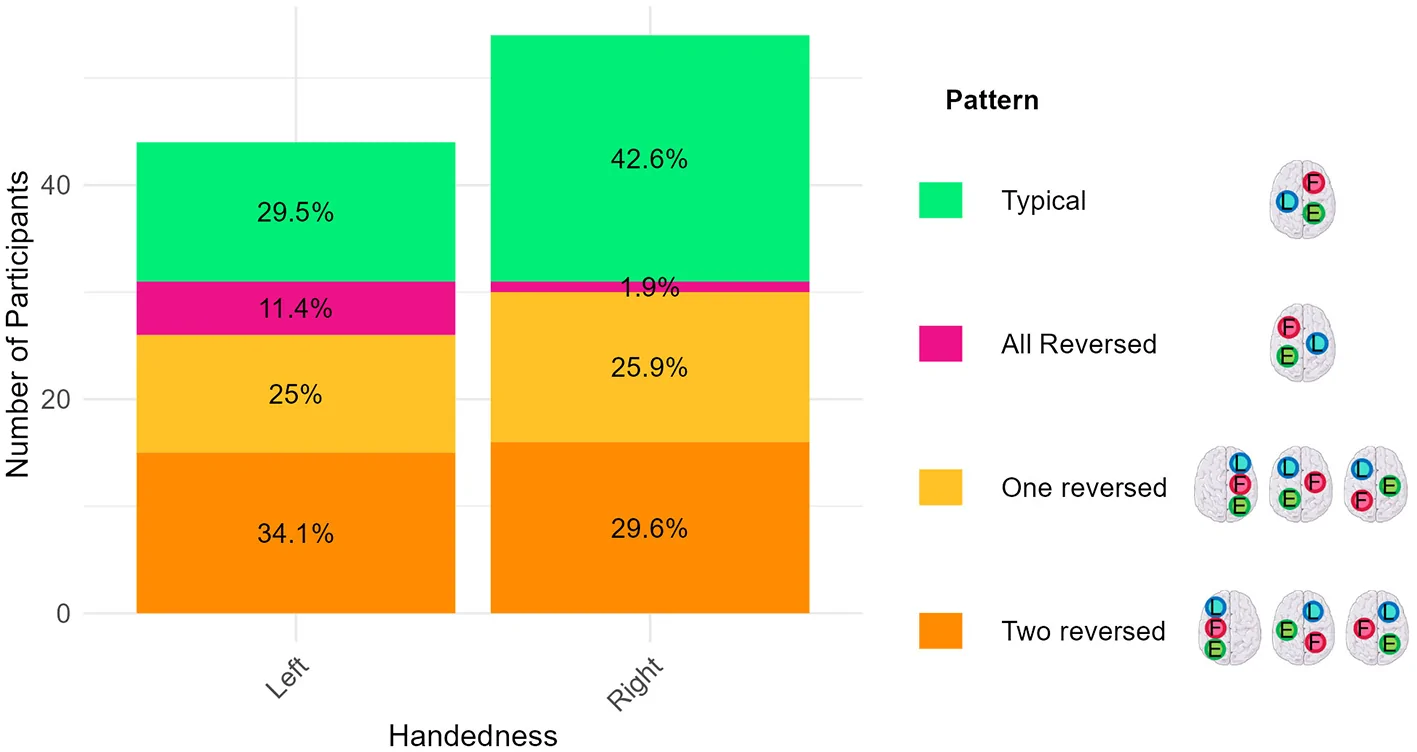
Patterns of hemispheric segregation for left- and right-handers. Handedness was determined using a cut-off of zero on the EHI.

To investigate whether there are significant differences between all segregation patterns within each handedness group, chi-square tests were computed for left- and right-handed individuals separately. Left-handers did not show significant differences between all segregation patterns (*X*^2^ = 5.09, *p* = 0.171). Right-handers, on the other hand, showed significant differences between the patterns (*X*^2^ = 18.74, *p* < 0.001). *Post-hoc* tests showed that this difference was most likely driven by the difference between rTFS and 1rFS (*p*_*adj*_ = 0.004), 2rFS (*p*_*adj*_ = 0.002), and typical (*p*_*adj*_ < 0.001). No differences between typical, 1rFS, and 2rFS were found. Individual cell-counts for each of the sub-groups can be found in Table 1.

**Table 1:** Number of participants showing each segregation pattern, by handedness group (left- and right-handed); values are counts and N denotes the total number of participants per group.

Handedness (2 groups)	*N*	TFS	1rFS	2rFS	rTFS
Left	44	13	11	15	5
Right	54	23	14	16	1

### 3.4 Hemispheric segregation by handedness (left, right, and mixed)

Separating the sample into left-, right-, and mixed-handed participants shows slightly different patterns of hemispheric segregation across all three groups, which, however, failed to get statistically significant [*X*^2^ = 9.16, *p* = 0.162*, V* = 0.13, 95% CI (0.00, 0.17)]. As can be seen in Figure 4 the percentage of TFS was 40.0% in the right-handed group, 48.4% in the mixed -handed group, but only 18.5% in the left -handed group. Across all groups, rTFS is the least common pattern, with only 2.5% of right-handers showing this pattern. Interestingly, left-handers seem to show a relatively high percentage of 2rFS (40.7%). The number of participants in each of the groups can be found in Table 2. Although the overall chi-square test did not reach statistical significance, inspection of SRM suggested a tendency for left-handers to show TFS less often than expected (18.5%; SRM = −2.31).

**Figure 4:**
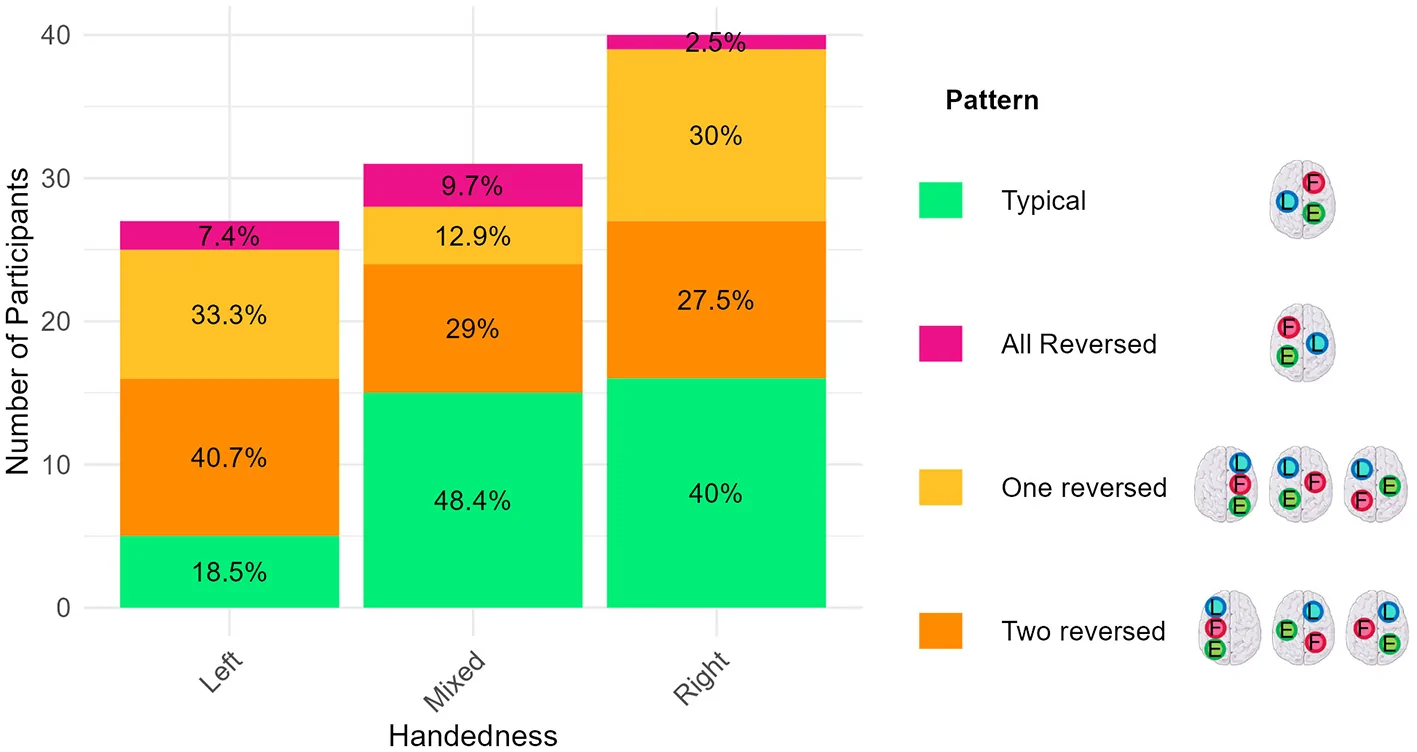
Patterns of hemispheric segregation for left-, mixed-, and right-handed participants.

**Table 2:** Number of participants showing each segregation pattern, by handedness group (left-, mixed-, and right-handed); values are counts and N denotes the total number of participants per group.

Handedness	*N*	TFS	1rFS	2rFS	rTFS
Left	27	5	9	11	2
Mixed	31	15	4	9	3
Right	40	16	12	11	1

Investigating the segregation patterns of each group separately shows no significant differences between all patterns for left-handers (*X*^2^ = 7.22, *p* = 0.068). For the right-handers (*X*^2^ = 12.2, *p* = 0.007) and mixed-handers (*X*^2^ = 11.71, *p* = 0.007) significant differences were found. Within the group of right-handers, there seem to be differences between the number of occurrences of rTFS and TFS (*p*_*adj*_ = 0.004), 1rFS (*p*_*adj*_ = 0.015), and 2rFS (*p*_*adj*_ = 0.026) functions. No differences were found between the TFS, 1rFS, and 2rFS functions. For mixed-handers, only the difference between rTFS and TFS survived correction for multiple comparisons (*p*_*adj*_ = 0.040).

### 3.5 Hemispheric segregation by sex

Investigating whether males and females show diverging patterns of hemispheric segregation revealed no significant differences between both sexes [*X*^2^ = 1.21, *p* = 0.765, *V* = 0.00, 95% CI (0.00, 0.25)]. As can be seen in Figure 5 females showed about 41.5% and males 31.1% of TFS. In both groups, the rTFS pattern is the rarest, with 5.7% of females and 6.7% of males showing this pattern. The number of participants in each of the groups can be found in Table 3.

**Figure 5:**
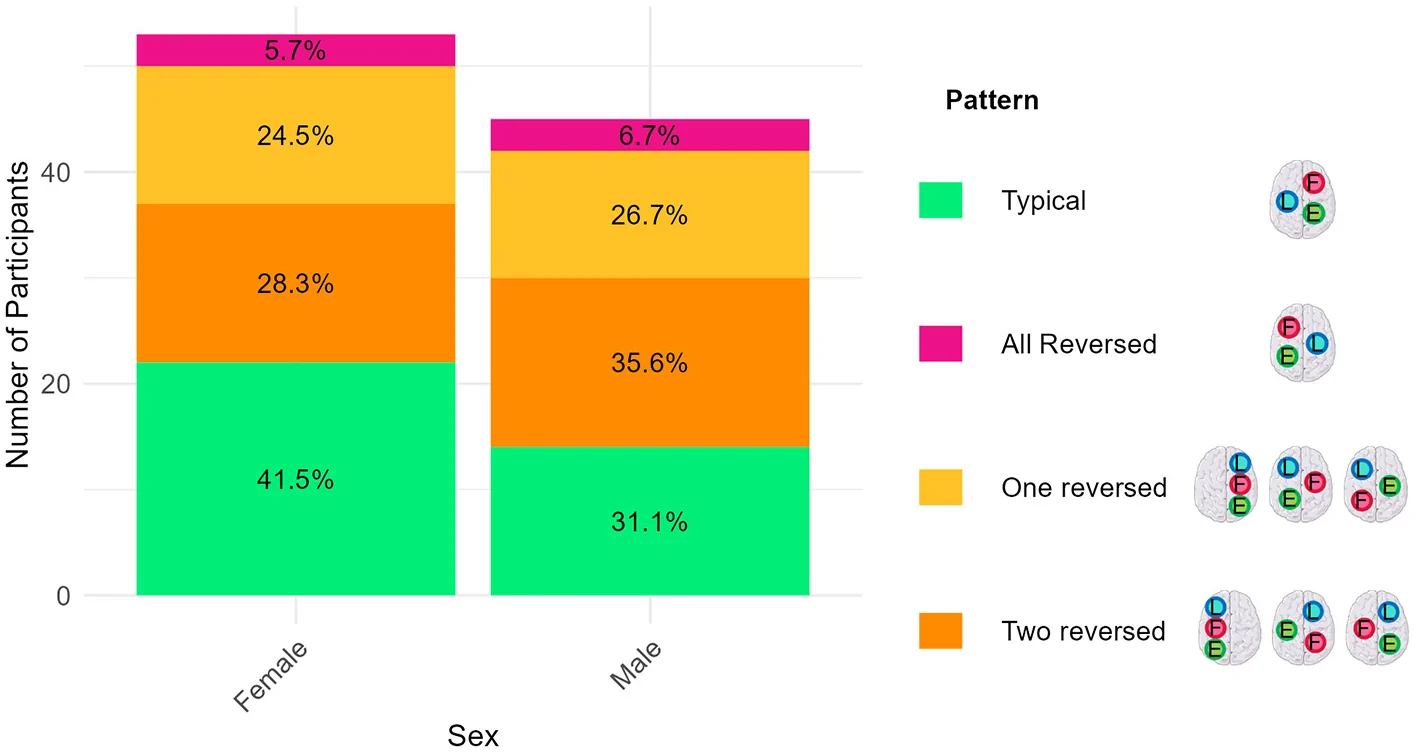
Patterns of hemispheroc segregation for females **(left)** and males **(right)**.

**Table 3:** Number of participants showing each segregation pattern, by sex; values are counts and N denotes the total number of participants per group.

Sex	*N*	TFS	1rFS	2rFS	rTFS
Female	53	22	13	15	3
Male	45	14	12	16	3

Investigating the segregation patterns within each group shows that females displayed significant differences between the segregation patterns (*X*^2^ = 13.94, *p* = 0.003), with significant differences between the rTFS and TFS (*p*_*adj*_ = 0.001), as well as between rTFS and 2rFS (*p*_*adj*_ = 0.046). A similar pattern was also observed in males (*X*^2^ = 8.78, p = 0.032), however, only the comparison between 2rFS and rTFS survived correction for multiple comparisons (*p*_*adj*_ = 0.027).

### 3.6 Hemispheric segregation by sex and handedness

Investigating the relationship between handedness and hemispheric segregation separately for males and females showed no significant effects for the males [*X*^2^ = 11.66, *p* = 0.065, *V* = 0.25, 95% CI (0.00, 0.44)]. However, for the females the test reached significance [*X*^2^ = 12.87, *p* = 0.036, *V* = 0.26, 95% CI (0.00, 0.42)]. Residual analysis of the females shows that these differences seem to be driven by the mixed-handed females, who tend to show more often than expected the TFS (66.7%; RSM = 3.01) and less often than expected the 1rFS pattern (9.5%; RSM=-2.06). As can be seen in Figure 6, the proportion of TFS in males was 50%, while only 15.4% and 10.0% of the left-handed and mixed-handed males, respectively, showed a typical pattern of segregation. For the left- and mixed-handed males a pattern of 2rFS seemed to be the most common, with 46.2% (left-handers) and 50.0% (mixed-handers). The number of participants in each of the groups can be found in Table 4. The residual analysis however, only suggested a slightly higher proportion of TFS in right-handers (50.0%; RSM = 2.68).

**Figure 6:**
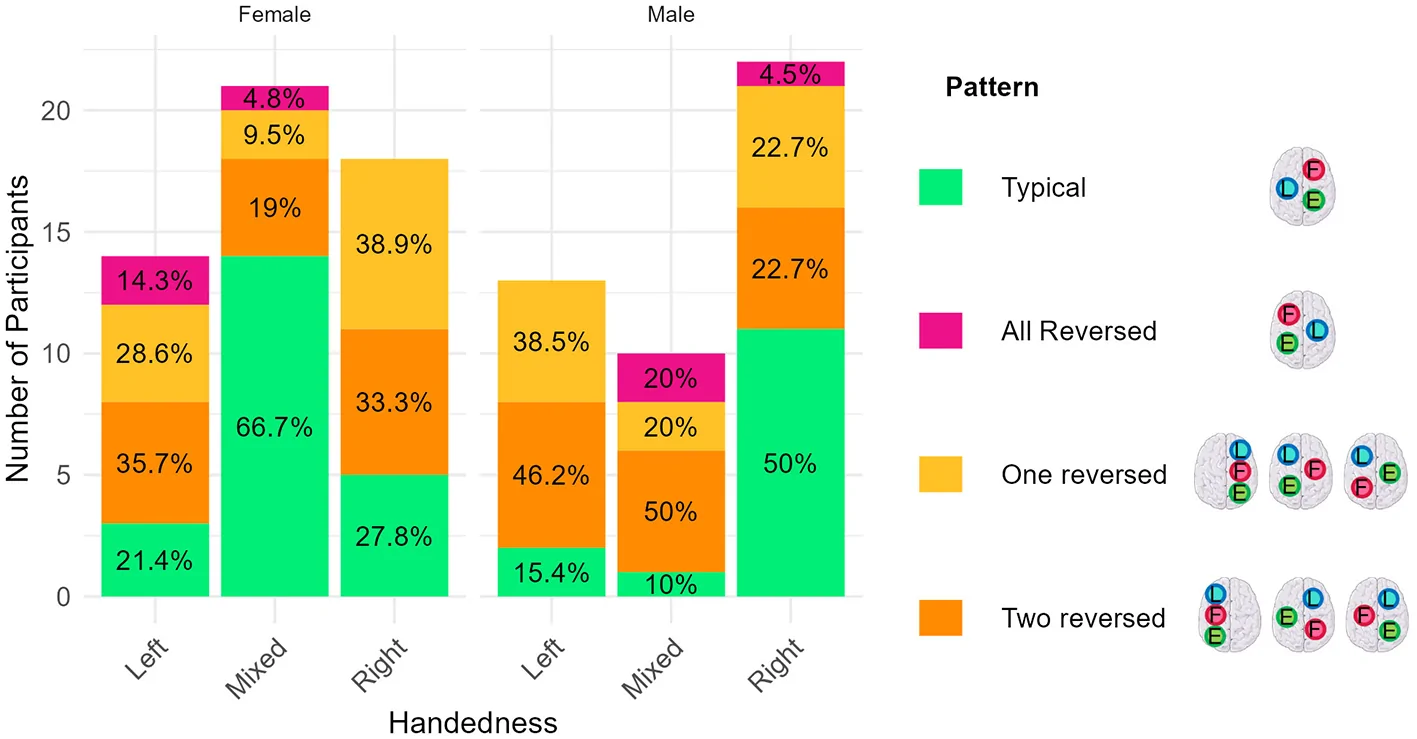
Patterns of hemispheric segregation for left-, mixed-, and right-handed females **(left)** and males **(right)**.

**Table 4:** Number of participants showing each segregation pattern, by sex and handedness; values are counts and N denotes the total number of participants per subgroup.

Sex	Handedness	*N*	TFS	1rFS	2rFS	rTFS
Female	Left	14	3	4	5	2
Male	Left	13	2	5	6	0
Female	Mixed	21	14	2	4	1
Male	Mixed	10	1	2	5	2
Female	Right	18	5	7	6	0
Male	Right	22	11	5	5	1

The within-group chi-square test revealed significant differences between segregation patterns for right-handed males (*X*^2^ = 9.27, *p* = 0.026) which was mostly driven by the difference between TFS and rTFS (*p*_*adj*_ = 0.046). For left-handed males (*X*^2^ = 7.00, *p* = 0.068) and mixed-handed males (*X*^2^ = 3.60, *p* =0.434) no significant differences were found, even though it is noticeable that no left-handed male showed the rTFS pattern. For the females, no differences in segregation patterns were found for right-handers (*X*^2^ = 6.44, *p* = 0.108) and left-handers (*X*^2^ = 1.43, *p* = 0.819). For mixed-handed females, a significant effect was found (X^2^ = 20.33, *p* < 0.001) which was mostly driven by the significant difference between rTFS and TFS (*p*_*adj*_ = 0.007).

### 3.7 Hemispheric segregation by handedness and sex across all patterns

Given the high proportion of the patterns with 1rFS or 2rFS, we investigated the hemispheric segregation across all possible patterns. Figure 7 suggests subtle group differences regarding which specific functions are reversed within the 1rFS and 2rFS patterns. In right- and mixed-handed males, the 2rFS pattern seems to be solely driven by the “emotional faces and faces reversed” pattern (all functions crowding in the left hemisphere). In contrast to that, left-handed males showed all three possible patterns of 2rFS (7.7% language and faces; 30.8% emotional faces and faces; 7.7% emotional faces and language reversed), however, with “crowding” in the left hemisphere being also more common than both other patterns. For the 1rFS pattern the picture is more diverse for the male participants, however, the pattern of reversed language lateralization is the most common across all three handedness groups (9.1% in right-handers; 20% in mixed-handers; 23.1% left-handers). For the females, the most common 2rFS pattern is a pattern of reversed face and emotional faces lateralization (all functions crowding in the left hemisphere), which is shown by all three handedness groups. Atypical face and language lateralization is only shown by right-handed females (5.6%) and atypical emotional faces and language lateralization only by left-handed females (14.3%). Across the 1rFS patterns, an atypical language lateralization is the most common pattern (all functions crowding in the right hemisphere), which is shown by 27% of right-handed, 4.8% of mixed-handed, and 7.1% of left-handed women. The number of participants in each of the groups can be found in Table 5.

**Table 5:** Number of participants showing each fine-grained segregation pattern, by sex and handedness. Values are counts; N denotes the total number of participants per subgroup.

Sex	Handed-ness	*N*	TFS	Only language reversed	Only faces reversed	Only emotional faces reversed	Faces & emotional faces reversed	Faces & language reversed	Emotional faces & language reversed	rTFS
Female	Left	14	3	1	0	3	3	0	2	2
Male	Left	13	2	3	0	2	4	1	1	0
Female	Mixed	21	14	1	1	0	4	0	0	1
Male	Mixed	10	1	2	0	0	5	0	0	2
Female	Right	18	5	5	0	2	5	1	0	0
Male	Right	22	11	2	1	2	5	0	0	1

**Figure 7:**
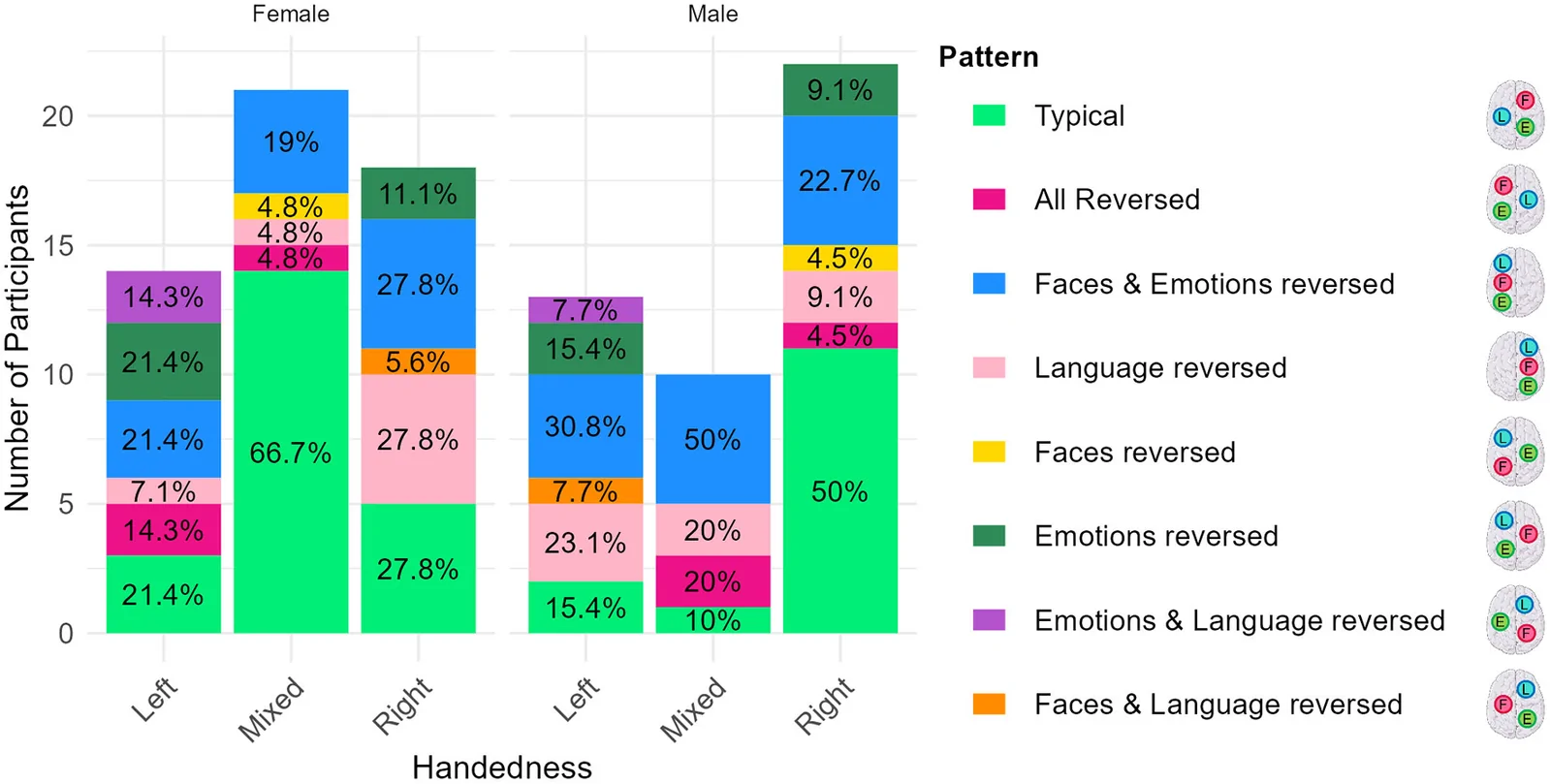
Percentages of all possible segregation patterns across left-, mixed-, and right-handed females **(left)** and males **(right)**.

Investigating differences between the handedness groups separately for each of the sexes, shows no differences in functional segregation between right-, left-, and mixed-handed males [*X*^2^ = 18.98, *p* = 0.125, *V* = 0.23, 95% CI (0.00, 0.34)]. However, the residual analysis suggested a slightly heightened proportion of TFS in right-handed males (50.0%; RSM = 2.68). The chi-square test for the females on the other hand did reach significance [*X*^2^ = 26.23, *p* = 0.007, *V* = 0.34, 95% CI (0.00, 0.45)]. The residual analysis showed that this was due to left-handed females showing more often than expected a pattern where emotional faces and language are atypically lateralized (14.3%; RSM = 2.41), mixed-handed females showing more often than expected a TFS pattern (66.7%; RSM = 3.01), and right-handed females having a heightened proportion of reversed language lateralization (27.8%; RSM = 2.25).

## 4 Discussion

The current study was (to our knowledge) the first study which used EEG to investigate hemispheric segregation. The goal of the study was to investigate patterns of hemispheric segregation across groups of sex (female and male) and handedness (left-, mixed-, and right-handed). Therefore, Gerrits (2024) model of hemispheric segregation was applied to early ERP components (N1/N170) of three different tasks, using an openly available dataset (Reinke et al., 2026). Investigating the lateralization patterns of each task separately, we found varying patterns between male and female left-, mixed-, and right-handers. Only male right-handers and female mixed-handers showed the expected higher proportion of typical compared to atypical lateralization during the words task (hypotheses 1 + 2). For no other task, or handedness group significant differences between typical and atypical lateralization was found. Interestingly, most participants showed at least a trend in the expected direction across all tasks. However, mixed-handed males showed a heightened proportion of atypical lateralization during the faces (70.0%) and emotional faces (70.0%) task. The same was true for left-handed females (71.4%) and left-handed males (53.8%) during the emotional faces task.

Regarding the functional segregation, we found that TFS is the most common pattern with 36.7% (3rd hypothesis), however, with less difference to all other segregation patterns than was expected. Furthermore, we found all possible patterns of segregation, highlighting that atypical lateralization is not a special case but rather a part of normal variability, which is in line with findings of high variability in segregation patterns by Gerrits et al. (2020). Applying our findings to Gerrits (2024) segregation bias model, we however found larger deviations from the predictions of the model than previous studies using fMRI or behavioral tasks. As explained above, the segregation bias model assumes that people either have a “typical” or “reversed typical” bias, with the “reversed bias” being very rare across the population. Predictions from the model were, that rTFS would being the rarest pattern across all possible combinations of hemispheric segregation (5th hypothesis) and that the 1rFS pattern will be more prevalent than the 2rFS pattern (4th hypothesis). However, a complete reversal of asymmetries was found in 6.1% of participants and is therefore not the least common pattern. A split-up of processing emotional and neutral faces, especially with a reversed language lateralization occurred less often than the complete reversal. Furthermore, the 2rFS pattern was the 2nd most common pattern (reversal of face and emotional faces) with 26.5% of participants showing this crowding of functions in the left hemisphere. These deviations may largely be explained by the composition of our sample. In the segregation bias model, handedness is itself one of the lateralized functions, and the model's predictions refer to a population with the typical distribution of handedness, in which a TFS bias is assumed for approximately 99% of individuals and left-handedness occurs in about 10% (Gerrits, 2024). Our sample, in contrast, contained a considerably higher proportion of non-right-handers (27.55% left-handed, 31.63% mixed-handed). Since atypical handedness is, within the model, associated with an increased likelihood of an rTFS bias, our sample is expected to be enriched for individuals with such a bias, which would increase the prevalence of reversed and crowded patterns. Descriptively, this is mirrored in our data: when considering right-handers separately, the ordering is at least numerically consistent with the model's predictions (TFS 40%, 1rFS 30%, 2rFS 27.5%, rTFS 2.5%), whereas this ordering is not preserved in the sample as a whole. It should be noted, however, that none of these differences reached statistical significance, and the observed proportions deviate considerably from the model's quantitative predictions even among right-handers.

Next to the segregation bias model, two more models have been proposed in order to explain how different functions lateralize to either the left or right hemisphere: the statistical hypothesis and the causal hypothesis. In the causal hypothesis it is assumed that the lateralization of one function forces other functions to the opposite hemisphere (Badzakova-Trajkov et al., 2016), while the statistical hypothesis states that each function has its own independent bias to either lateralize to the left or the right hemisphere (Badzakova-Trajkov et al., 2016). Applying both ideas to our data would mean, that if lateralization happens as proposed by the causal hypothesis most people should show TFS and rTFS would be the only other pattern of lateralization. This is because if e.g., language is lateralized to the left it should “push” face and emotional face processing to the right and if language is lateralized to the right, it should “push” face and emotional face processing to the left. This, however, is not what we observed in our data, as crowding of all functions in one hemisphere are the 2nd and 3rd most common patterns within our data. Furthermore, the colocalization of language and faces appears to be quite common, as 45.9% of participants show a segregation pattern, where language and face processing are located within the same hemisphere. With the statistical hypothesis, there should be no correlation between the lateralization of the different functions, which is partly in line with what we found. However, this would not explain why face and emotional face perception lateralize to the same hemisphere in 83.6% of participants. Both of these conclusions rest on the assumption that the three functions examined here constitute the relevant system. If further lateralized functions are considered, such as visuospatial attention, which Gerrits (2024) includes as a third function in his model, the observed crowding would not necessarily contradict the causal hypothesis. An unmeasured function lateralized to one hemisphere could in principle push language, face, and emotional face processing jointly to the other, producing exactly the co-localization we observe. The same applies to the statistical account. Gerrits (2024) segregation bias model, which essentially implements a statistical hypothesis, predicts that when two functions are already lateralized atypically, any further function becomes substantially more likely to follow. The crowding observed here is therefore compatible with both accounts once more than three functions are considered, and our data cannot adjudicate between them.

Returning to the co-lateralization of face and emotional face processing, given the high similarity between the face and emotional face stimuli, this co-lateralization seems rational, and it could be argued that emotional and neutral faces underlie the same cognitive process. Given this close relationship, we refer to “emotional faces” throughout, to make explicit that our findings concern the processing of emotion in the context of faces rather than emotion processing in general. This argument, however, could not account for the observed “split-up” (functions being lateralized to different hemispheres) of face and emotional faces which was seen in 16.3% of participants, which might indicate that processing emotions modulates the hemispheric asymmetries during face processing. Evidence for such a modulation at the level of the N170 is mixed, which fits the fact that only a minority of our participants showed a split-up. A meta-analysis of 57 experiments found the N170 to be sensitive to facial expressions, but these effects were not associated with a consistent lateralization pattern (Hinojosa et al., 2015). Individual studies, however, have reported hemisphere-specific effects. Stekelenburg and de Gelder (2004) found an interaction between emotional expression and recording site, with fearful faces eliciting a more negative N170 than neutral faces at P7 but not at P8. Such a selective enhancement over the left hemisphere would reduce the relative rightward asymmetry for emotional faces and could, in participants with weak asymmetries, shift the classified direction of lateralization. This finding stems from a single study with a small sample and concerned fearful expressions only, but it illustrates the kind of inconsistency that the meta-analysis summarizes, and it offers a plausible mechanism for the divergence we observe in a subset of participants. Three important and competing theories of lateralization in emotion processing are the right hemisphere hypothesis (RHH), approach-withdrawal hypothesis and the valence theory (Davidson, 1984; Demaree et al., 2005). The RHH postulates that all emotions are processed in the right hemisphere, while the valence theory suggests that the right hemisphere is relevant for processing negative emotions while the left hemisphere is more involved in processing positive emotions. The approach-withdrawal hypothesis on the other hand locates withdrawal related emotions (e.g., sadness and disgust) in the right hemisphere while approach related emotions (e.g., happiness and anger) are thought to be processed by the left hemisphere. The current dataset used 80 smiling faces, 40 angry faces, and 40 fearful faces. Depending on which hypothesis underlies emotional lateralization this would either mean that there should be a strong rightward lateralization for all 160 stimuli (RHH), that the 80 positive (lateralized to the left) and 80 negative faces (lateralized to the right) would lead to an overall lateralization around zero (valence hypothesis), or that the 120 approach related emotions (happiness and anger) would lead to an overall left lateralization, due to only 40 withdrawal (fear) related emotions being presented (approach-withdrawal hypothesis). Even though different influences of emotions might be possible, it is important to notice that the original paper providing the current dataset (Reinke et al., 2026) did not find a modulation of asymmetries by emotional valence. Across all segregation patterns, emotion was lateralized to the left in 44.9% of participants, suggesting a variable pattern across participants. Importantly, with the approach chosen in this study, we cannot identify participants with an almost symmetrical pattern, as we did not assess the strength of the lateralization. An almost symmetrical pattern, however, might very well lead to the occurrence of half of the participants showing a tendency toward the right and the other half a tendency toward the left (as the cut-off was zero). Another aspect that might lead to the different lateralization pattern of the emotional faces task might lie in different strategies used by the participants. The underlying task did not require an emotion identification by the participants, rather they were instructed to respond to all female faces. Therefore, some participants might have totally ignored the emotional valence, leading to a stronger face-related right lateralization, while others might have consciously perceived the different emotions, leading to a possible left-shift due to the higher proportion of approach related emotions (see above). Such strategy-dependent variation is plausible given that the N170 is not a purely automatic response but is modulated by attention and task demands (Dou et al., 2021). Whether such attentional differences also shift the direction of lateralization, rather than only its magnitude, remains speculative, but they might provide a plausible source of the individual variability we observed.

Regarding the influence of handedness (6th hypothesis) we found no difference between left- and right-handers. However, only right-handers showed a significantly higher proportion of TFS compared to rTFS, which was not found in left-handers. The same was true when handedness was assessed in three categories (left-, mixed-, and right-handers). No significant differences in lateralization patterns were found between all three groups, however, only in right-handers rTFS occurred significantly less often all other segregation patterns, while in mixed-handers only the difference between TFS and rTFS remained significant. In left-handers no differences between all four segregation patterns were found. This implies that there are slight differences between different handedness groups, however, comparing the segregation patterns – especially between mixed- and right-handed individuals – hints toward the absence of a linear gradient from right- to left-handedness. This would imply that hemispheric segregation is not merely a function of handedness direction, but underlying processes are more complex then assumed when investigating handedness as a binary measure.

We also were not able to find significant differences between both sexes regarding their segregation pattern (7th hypothesis). However, contrary to our expectations, females showed more often TFS (41.5%) compared to males (31.1%). Also, only females showed significantly higher proportions of TFS, as well as 2rFS, compared to rTFS. For males, only the comparison between 2rFS and rTFS got significant. We based our hypothesis regarding sex differences on studies reporting that females tend to be less strongly lateralized than males, an effect that is small but robust (Hirnstein et al., 2019). Early perceptual EEG research suggests, however, that this effect is domain-specific rather than uniform. For faces, the N170 is typically right-lateralized in males but more bilateral in females, a difference that is well replicated (Proverbio et al., 2010; Proverbio, 2021). For written words, in contrast, a large-scale investigation found very little evidence for sex differences in reading lateralization (Chiarello et al., 2009). Notably, the more bilateral face processing reported for females would, if anything, predict a lower rather than a higher proportion of typical segregation in women, since typical segregation requires a rightward asymmetry for faces. The higher aggregate proportion of TFS in females therefore runs counter to what this literature would lead one to expect. Two considerations may account for this. First, we measured only the direction, not the strength of lateralization. Weak but consistent rightward asymmetry in females would be classified as typical just as a stronger lateralization would, meaning that a more bilateral amplitude pattern does not necessarily translate into more atypical classifications. Second, as we detail below, this sex difference is largely driven by mixed-handed females and does not reflect a uniform sex effect.

Combining handedness and sex revealed sex-specific influences of handedness on segregation patterns. While statistically left-, mixed-, and right-handed males did not differ in their segregation patterns, mixed-handed females were significantly different from left- and right-handed females. It was found that mixed-handed females tended to show more often than expected the TFS pattern (66.7%) and less often the 1rFS pattern (9.5%). Within each group, only male right-handers and female mixed-handers showed the expected higher proportion of TFS compared to rTFS. Even though, statistically not significant, it is noticeable that especially mixed-handed (10.0%) and left-handed (15.4%) males show a very low proportion of TFS, hinting toward handedness having an influence on hemispheric functional segregation also in this population. Especially intriguing is the finding that mixed-handed females tend to show very high levels of TFS. Since mixed-handed females are a group of participants that have been largely neglected in research so far, this highlights the importance to include a wide variety of participants (especially in asymmetry research) and carefully consider the effects of sex and handedness in a more fine-grained manner than before. This is highlighted also through the fact, that especially in the mixed-handed population, males and females tend to exhibit vastly different segregation patterns, which were covered up before when investigating the effect of handedness across males and females. Also, the finding of higher proportions of TFS in right-handers seems to only be true for the male participants, not females.

As the proportions of 1rFS and 2rFS were rather large within our sample, we also investigated sex and handedness related differences across all possible segregation patterns. However, as there are eight possible combinations, interpreting differences between all groups is challenging. What is noticeable is that a reversed lateralization of emotional faces and language was only seen in female (14.3%) and male (7.7%) left-handers. While three participant groups seem to have one pattern that dominates like for example TFS in mixed handed females (66.7%) and right-handed males (50.0%) and reversed emotional faces and face processing in mixed handed males (50.0%), the other three groups seem to have a relatively mixed pattern, which is especially obvious in left-handed females (see Figure 7) where the percentages for each pattern only vary between 7.1% (language reversed) and 21.4% (typical, emotional faces reversed, and faces and emotional faces reversed all being present in 21.4% of participants). Besides that, the patterns seem to be more or less randomly distributed across all groups with no pattern becoming apparent, making it difficult to interpret.

Compared to previous studies investigating segregation patterns using fMRI (Gerrits et al., 2020) or within sensory-motor domains (Marcori et al., 2026), the present study focused on segregation patterns derived from the early N1 component of the EEG. By doing so, we specifically targeted early perceptual processing stages rather than later, higher-level cognitive processes. This difference in temporal sensitivity may contribute to deviations between our findings and previous studies, as well as the segregation bias model proposed by Gerrits (2024) which was primarily based on fMRI data. While fMRI captures neural activity integrated over several seconds and is therefore more sensitive to later, integrative stages of processing, early ERP components such as the N1 reflect relatively rapid perceptual processes occurring within the first 200 ms after stimulus onset. It should be noted that the posterior sites we averaged across span a range of the ventral visual stream, from lateral occipital (O1/O2) to occipitotemporal locations (P7/P8), which have been associated with somewhat different aspects of face and word processing. Our claim, however, concerns the timing rather than the anatomical locus of segregation. All sites were quantified within the same early time window, during which activity at each of them reflects early perceptual rather than higher-order processing. As a result, EEG-based lateralization indices may not only reflect hemispheric dominance for specific cognitive functions, but also more basic differences in perceptual processing styles. In this context, hemispheric asymmetries have often been linked to differences in processing styles, with the right hemisphere being more strongly involved in holistic and configural processing, and the left hemisphere contributing more to local, sequential, and analytical processing (Fink et al., 1996). Functional lateralization may therefore emerge not only because a cognitive function is tied to a specific hemisphere, but also because successful task performance benefits from a particular processing style. As the tasks used in the present study were relatively simple recognition-based paradigms that did not require extensive semantic or strategic processing, hemispheric asymmetries may have been less pronounced than in studies employing more cognitively demanding paradigms. Under such conditions, individual differences in preferred processing strategies may become more influential, leading some participants to rely more strongly on analytical processing, while others may adopt a more holistic processing approach. Such individual differences in processing style may help explain why patterns reflecting “crowding” of functions within one hemisphere -either left or right- were among the most common segregation patterns in the present sample. Beyond the functional similarities between faces and emotional faces discussed above, hemispheric crowding may therefore also reflect a general bias toward either analytical or holistic processing. Individuals showing left-hemispheric crowding may rely more strongly on feature-based or sequential strategies, whereas individuals showing right-hemispheric crowding may preferentially engage configural or integrative processing styles.

Importantly, these findings should not be interpreted as contradicting the segregation bias model proposed by Gerrits (2024). Rather, they suggest that hemispheric segregation may manifest differently depending on the methodological approach and the stage of processing being examined. EEG captures neural activity at a very early time point within an extended processing cascade, whereas fMRI reflects temporally integrated activation patterns that likely include later cognitive and decisional processes. The present results therefore extend the segregation bias model by demonstrating that patterns of functional segregation can already be observed at early perceptual stages, while also highlighting that hemispheric organization may be influenced by both functional specialization and individual differences in processing style. Integrating findings across methodological approaches may therefore provide a more comprehensive understanding of hemispheric segregation across the full temporal course of cognitive processing.

## 5 Limitations

Even though we carefully executed all analysis and interpretations of our results, some limitations of our study have to be taken into consideration. First of all, the sample sizes within each group are very small (e.g., especially for the mixed-handed males). These small sample sizes might distort the results and lead to an overestimation of atypical segregation patterns. Furthermore, the high number of statistical tests increases the risk of Type I error and reduces power after correction, meaning that some genuine group differences may not have reached significance in our analysis. Relatedly, the confidence intervals for our effect sizes were wide and, in several cases, included zero even for significant effects. This indicates that while our tests show that the distribution of segregation patterns deviates from chance, the magnitude of these associations could not be estimated with precision. Significant results should therefore be interpreted as evidence that an association exists, rather than as reliable estimates of its strength. The choice of measuring only the direction of laterality and not the strength is another important point. Non-lateralized individuals were “forced” into either the left- or right-dominant group, possibly increasing the number of atypical lateralization and therefore segregation patterns. In previous papers the definition of cut-off points to distinguish lateralized from non-lateralized individuals has been discussed (Vingerhoets et al., 2023; Gerrits and Vingerhoets, 2025), but no final decision has been made so far. Importantly, the EEG is very susceptible to environmental noise as well as muscle artifacts, which increases uncertainty to the measurement. This is especially evident in the fact, that we had to use a different measure of the N1 for the language task (mean) compared to the faces and emotional faces task (peak-to-peak; for more information see the methods section as well as Reinke et al., 2026). Given the very large and asymmetric P1 component for the words task, choosing the same approach as for the faces/emotional faces task (peak-to-peak) would distort the N1-asymmetries, leading to the absence of the very well-established left-hemispheric pattern.

## 6 Outlook

The current finding of differences between left-, mixed-, and right-handers that became particularly pronounced when males and females were analyzed separately, further emphasize that handedness should be conceptualized as a continuous rather than a binary trait. Considering both handedness and sex as graded or interacting sources of variability may provide a more comprehensive account of individual differences in hemispheric segregation. Rather than reflecting noise, such variability may point toward underlying biological and developmental factors that shape the emergence and strength of lateralization. Future research would therefore benefit from systematically examining which factors modulate hemispheric segregation patterns, including sex-related influences, developmental trajectories, and potentially genetic or experiential contributions. Furthermore, hemispheric asymmetries are often altered in clinical population (Mundorf and Ocklenburg, 2021; Ocklenburg et al., 2024; Packheiser et al., 2025). Therefore, assessing segregation patterns in clinical populations might also further increase our understanding of factors that influence the segregation or crowing of different functions between or within one hemisphere.

Within this framework, an important question concerns how different cognitive functions relate to each other in their lateralization patterns. The current findings further highlight the possibility that certain functions may tend to co-lateralize within individuals (e.g., face and emotional face processing), while others may lateralize more independently or even show complementary hemispheric distributions. Mapping such patterns of co-lateralization across domains could provide deeper insight into the organizational principles governing hemispheric specialization. At the same time, inconsistencies across studies suggest that the observed degree of lateralization may also depend on when and how asymmetries are measured. Temporal dynamics, task characteristics, and methodological differences may influence the detectability and apparent strength of hemispheric asymmetries. A more systematic consideration of measurement timing and operationalization of asymmetry may therefore help to disentangle genuine interindividual variability from methodological variance. Future studies should therefore aim to investigate for example EEG, fMRI, and behavior simultaneously to assess functional hemispheric segregation patterns across all three levels to see how segregation patterns align (or not align) across measurements.

Finally, a more continuous view of lateralization also requires a clearer conceptualization of what constitutes non-lateralization. Rather than treating it as a residual category, future work should aim to distinguish between truly bilateral processing, weak or inconsistent lateralization, and patterns that appear non-lateralized due to measurement constraints. Refining these definitions will be essential for developing a more integrative account of hemispheric organization that captures the full spectrum of individual variability.

## Data Availability

Publicly available datasets were analyzed in this study. This data can be found here: the analysis was conducted using an openly available dataset from OpenNeuro (OpenNeuro Dataset ds007172). The dataset was produced and uploaded by our group and a full description of all tasks can be found in Reinke et al. (2026). The preprocessed data, as well as the data on handedness can be found in the OSF: https://osf.io/g74vq?view_only=cc40b6db1a9d4745b70164d417fbe731.

## References

[B1] Amora K. K. Tretow A. Verwimp C. Tijms J. Leppänen P. H. T. Csépe V. . (2022). Typical and atypical development of visual expertise for print as indexed by the visual word N1 (N170W): a systematic review. Front. Neurosci. 16:898800. doi: 10.3389/fnins.2022.898800 PMC9279737 35844207

[B2] Badzakova-Trajkov G. Corballis M. C. Häberling I. S. (2016). Complementarity or independence of hemispheric specializations? a brief review. Neuropsychologia 93, 386–393. doi: 10.1016/j.neuropsychologia.2015.12.018 26712091

[B3] Beraha E. Eggers J. Hindi Attar C. Gutwinski S. Schlagenhauf F. Stoy M. . (2012). Hemispheric asymmetry for affective stimulus processing in healthy subjects-a fMRI study. PLoS One 7:e46931. doi: 10.1371/journal.pone.0046931 PMC3466188 23056533

[B4] Bergsma W. (2013). A bias-correction for Cramér's and Tschuprow's. J. Korean Stat. Soc. 42, 323–328. doi: 10.1016/j.jkss.2012.10.002

[B5] Bisiacchi P. Cainelli E. (2022). Structural and functional brain asymmetries in the early phases of life: a scoping review. Brain Struct. Funct. 227, 479–496. doi: 10.1007/s00429-021-02256-1 PMC8843922 33738578

[B6] Boles D. B. (2005). A large-sample study of sex differences in functional cerebral lateralization. J. Clin. Exp. Neuropsychol. 27, 759–768. doi: 10.1081/13803390590954263 16019651

[B7] Bourne V. J. Maxwell A. M. (2010). Examining the sex difference in lateralisation for processing facial emotion: does biological sex or psychological gender identity matter? Neuropsychologia 48, 1289–1294. doi: 10.1016/j.neuropsychologia.2009.12.032 20036677

[B8] Broca P. (1861). Perte de la parole, ramollissement chronique et destruction partielle du lobe antérieur gauche du cerveau [Loss of speech, chronic softening, and partial destruction of the left anterior lobe of the brain]. Bulletins de la Société d'Anthropologie de Paris 2, 235–238.

[B9] Chiarello C. Welcome S. E. Halderman L. K. Towler S. Julagay J. Otto R. . (2009). A large-scale investigation of lateralization in cortical anatomy and word reading: are there sex differences? Neuropsychology 23, 210–222. doi: 10.1037/a0014265 PMC3086359 19254094

[B10] Civera T. Perea M. Leone-Fernandez B. Vergara-Martínez M. (2024). The effect of inter-letter spacing on the N170 during visual word recognition: an event-related potentials experiment. Cogn. Affect. Behav. Neurosci. 24, 1096–1108. doi: 10.3758/s13415-024-01221-9 PMC11525266 39313747

[B11] Davidson R. J. (1984). “Affect, cognition, and hemispheric specialization,” in Emotion, Cognition, and Behavior, eds. Izard C. E. Kagan J. Zajonc R. (New York, NY: Cambridge University Press), 320–365.

[B12] Demaree H. A. Everhart D. E. Youngstrom E. A. Harrison D. W. (2005). Brain lateralization of emotional processing: historical roots and a future incorporating “dominance”. Behav. Cogn. Neurosci. Rev. 4, 3–20. doi: 10.1177/1534582305276837 15886400

[B13] Dou H. Liang L. Ma J. Lu J. Zhang W. Li Y. . (2021). Irrelevant task suppresses the N170 of automatic attention allocation to fearful faces. Sci. Rep. 11:11754. doi: 10.1038/s41598-021-91237-9 PMC8175742 34083660

[B14] Emmorey K. Midgley K. J. Kohen C. B. Sehyr Z. S. Holcomb P. J. (2017). The N170 ERP component differs in laterality, distribution, and association with continuous reading measures for deaf and hearing readers. Neuropsychologia 106, 298–309. doi: 10.1016/j.neuropsychologia.2017.10.001 PMC5694363 28986268

[B15] Fajkowska M. Eysenck M. W. Zagórska A. Jaśkowski P. (2011). ERP responses to facial affect in low-anxious, high-anxious, repressors and defensive high-anxious individuals. Pers. Individ. Differ. 50, 961–976. doi: 10.1016/j.paid.2010.11.023

[B16] Fink G. R. Halligan P. W. Marshall J. C. Frith C. D. Frackowiak R. S. Dolan R. J. . (1996). Where in the brain does visual attention select the forest and the trees? Nature 382, 626–628. doi: 10.1038/382626a0 8757132

[B17] Frässle S. Paulus F. M. Krach S. Schweinberger S. R. Stephan K. E. Jansen A. . (2016). Mechanisms of hemispheric lateralization: Asymmetric interhemispheric recruitment in the face perception network. NeuroImage 124, 977–988. doi: 10.1016/j.neuroimage.2015.09.055 26439515

[B18] Gao L. Xu J. Zhang B. Zhao L. Harel A. Bentin S. . (2009). Aging effects on early-stage face perception: an ERP study. Psychophysiology 46, 970–983. doi: 10.1111/j.1469-8986.2009.00853.x 19558400

[B19] Gerrits R. (2024). Variability in hemispheric functional segregation phenotypes: a review and general mechanistic model. Neuropsychol. Rev. 34, 27–40. doi: 10.1007/s11065-022-09575-y 36576683

[B20] Gerrits R. Verhelst H. Vingerhoets G. (2020). Mirrored brain organization: statistical anomaly or reversal of hemispheric functional segregation bias? Proc. Natl. Acad. Sci. U S A 117, 14057–14065. doi: 10.1073/pnas.2002981117 PMC7322015 32513702

[B21] Gerrits R. Vingerhoets G. (2025). The reliability of bilateral cerebral laterality for word generation: Who is left in the middle? Imaging Neurosci. 3:IMAG.a.102. doi: 10.1162/IMAG.a.102 PMC12336058 40800965

[B22] Godard O. Fiori N. (2010). Sex differences in face processing: are women less lateralized and faster than men? Brain .Cogn. 73, 167–175. doi: 10.1016/j.bandc.2010.04.008 20621740

[B23] Gramfort A. Luessi M. Larson E. Engemann D. A. Strohmeier D. Brodbeck C. . (2013). MEG and EEG data analysis with MNE-Python. Front. Neurosci. 7:267. doi: 10.3389/fnins.2013.00267 PMC3872725 24431986

[B24] Grossi G. Savill N. Thomas E. Thierry G. (2010). Posterior N1 asymmetry to english and welsh words in early and late english-welsh bilinguals. Biol. Psychol. 85, 124–133. doi: 10.1016/j.biopsycho.2010.06.003 20542079

[B25] Gupta G. Pandey R. (2010). Assessment of hemispheric asymmetry: development and psychometric evaluation of a chimeric face test. Ind. Psychiatry J. 19, 30–36. doi: 10.4103/0972-6748.77632 PMC3105555 21694788

[B26] Haberman S. J. (1973). The analysis of residuals in cross-classified tables. Biometrics 29:205. doi: 10.2307/2529686

[B27] Hinojosa J. A. Mercado F. Carretié L. (2015). N170 sensitivity to facial expression: a meta-analysis. Neurosci. Biobehav. Rev. 55, 498–509. doi: 10.1016/j.neubiorev.2015.06.002 26067902

[B28] Hirnstein M. Hugdahl K. Hausmann M. (2019). Cognitive sex differences and hemispheric asymmetry: a critical review of 40 years of research. Laterality 24, 204–252. doi: 10.1080/1357650X.2018.1497044 29985109

[B29] Hirnstein M. Westerhausen R. Korsnes M. S. Hugdahl K. (2013). Sex differences in language asymmetry are age-dependent and small: a large-scale, consonant-vowel dichotic listening study with behavioral and fMRI data. Cortex 49, 1910–1921. doi: 10.1016/j.cortex.2012.08.002 22980918

[B30] Hugdahl K. Thomsen T. Ersland L. (2006). Sex differences in visuo-spatial processing: an fMRI study of mental rotation. Neuropsychologia 44, 1575–1583. doi: 10.1016/j.neuropsychologia.2006.01.026 16678867

[B31] Hugdahl K. Westerhausen R. (2016). Speech processing asymmetry revealed by dichotic listening and functional brain imaging. Neuropsychologia 93, 466–481. doi: 10.1016/j.neuropsychologia.2015.12.011 26706774

[B32] Kansaku K. Kitazawa S. (2001). Imaging studies on sex differences in the lateralization of language. Neurosci.Res. 41, 333–337. doi: 10.1016/S0168-0102(01)00292-9 11755219

[B33] Kimura D. (1967). Functional asymmetry of the brain in dichotic listening. Cortex 3, 163–178. doi: 10.1016/S0010-9452(67)80010-8

[B34] Larson E. Gramfort A. Engemann D. A. Leppäkangas J. Brodbeck C. Jas M. . (2026). MNE-Python. Zenodo. doi: 10.5281/zenodo.592483

[B35] Marcori A. J. Okazaki V. H. A. Ocklenburg S. (2026). Functional hemispheric segregation of sensory-motor laterality. Neuropsychologia 221:109327. doi: 10.1016/j.neuropsychologia.2025.109327 41314380

[B36] Mundorf A. Getzmann S. Gajewski P. D. Larra M. F. Wascher E. Genç E. . (2024). Phenotyping in clinical laterality research: a comparison of commonly used methods to determine mixed-handedness and ambidexterity. Laterality 29, 331–349. doi: 10.1080/1357650X.2024.2370871 38968414

[B37] Mundorf A. Ocklenburg S. (2021). The Clinical Neuroscience of Lateralization. London: Routledge. doi: 10.4324/9781003082507

[B38] Ocklenburg S. Güntürkün O. (2024). The Lateralized Brain: The Neuroscience and Evolution of Hemispheric Asymmetries. Elsevier. doi: 10.1016/B978-0-323-99737-9.00003-3

[B39] Ocklenburg S. Mundorf A. Gerrits R. Karlsson E. M. Papadatou-Pastou M. Vingerhoets G. . (2024). Clinical implications of brain asymmetries. Nat. Rev. Neurol. 20, 383–394. doi: 10.1038/s41582-024-00974-8 38783057

[B40] Oldfield R. C. (1971). The assessment and analysis of handedness: the Edinburgh inventory. Neuropsychologia 9, 97–113. doi: 10.1016/0028-3932(71)90067-4 5146491

[B41] Ortigue S. Thut G. Landis T. Michel C. M. (2005). Time-resolved sex differences in language lateralization. Brain 128:E28. doi: 10.1093/brain/awh386 15845626

[B42] Packheiser J. Borawski J. Berretz G. Merklein S. A. Papadatou-Pastou M. Ocklenburg S. . (2025). Handedness in mental and neurodevelopmental disorders: A systematic review and second-order meta-analysis. Psychol. Bull. 151, 476–512. doi: 10.1037/bul0000471 40310231

[B43] Packheiser J. Schmitz J. Berretz G. Carey D. P. Paracchini S. Papadatou-Pastou M. . (2020). Four meta-analyses across 164 studies on atypical footedness prevalence and its relation to handedness. Sci. Rep. 10:14501. doi: 10.1038/s41598-020-71478-w PMC7468297 32879356

[B44] Papadatou-Pastou M. Ntolka E. Schmitz J. Martin M. Munafò M. R. Ocklenburg S. . (2020). Human handedness: a meta-analysis. Psychol. Bull. 146, 481–524. doi: 10.1037/bul0000229 32237881

[B45] Prete G. Capotosto P. Zappasodi F. Tommasi L. (2018). Contrasting hemispheric asymmetries for emotional processing from event-related potentials and behavioral responses. Neuropsychology 32, 317–328. doi: 10.1037/neu0000443 29469582

[B46] Proverbio A. M. (2021). Sexual dimorphism in hemispheric processing of faces in humans: a meta-analysis of 817 cases. Soc. Cogn. Affect. Neurosci. 16, 1023–1035. doi: 10.1093/scan/nsab043 PMC8483282 33835164

[B47] Proverbio A. M. (2022). “*EEG and ERPs in the Study of Language and Social Knowledge,”* in Social and Affective Neuroscience of Everyday Human Interaction (Cham: Springer International *Publishing)* 195−212. doi: 10.1007/978-3-031-08651-9_12

[B48] Proverbio A. M. Brignone V. Matarazzo S. Del Zotto M. Zani A. (2006). Gender differences in hemispheric asymmetry for face processing. BMC Neurosci. 7:44. doi: 10.1186/1471-2202-7-44 PMC1523199 16762056

[B49] Proverbio A. M. Riva F. Martin E. Zani A. (2010). Face coding is bilateral in the female brain. PLoS One 5:e11242. doi: 10.1371/journal.pone.0011242 PMC2888585 20574528

[B50] Quin-Conroy J. E. Bayliss D. M. Daniell S. G. Badcock N. A. (2024). Patterns of language and visuospatial functional lateralization and cognitive ability: a systematic review. Laterality 29, 63–96. doi: 10.1080/1357650X.2023.2263199 37771079

[B51] R Core Team (2023). R: A Language and Environment for Statistical Computing. Vienna: R Foundation for Statistical Computing. Available online at: https://www.R-project.org/ (Accessed June 1, 2026).

[B52] Reinke P. Deneke L. Ocklenburg S. (2024). Asymmetries in event-related potentials part 1: a systematic review of face processing studies. Int. J. Psychophysiol. 202:112386. doi: 10.1016/j.ijpsycho.2024.112386 38914138

[B53] Reinke P. Deneke L. Ocklenburg S. (2026). Hemispheric asymmetries in the EEG: is there an association between N1 lateralization and alpha asymmetry? Laterality 31, 40–89. doi: 10.1080/1357650X.2025.2591660 41309445

[B54] Spironelli C. Angrilli A. (2007). influence of phonological, semantic and orthographic tasks on the early linguistic components N150 and N350. Int. J. Psychophysiol. 64, 190–198. doi: 10.1016/j.ijpsycho.2007.02.002 17363097

[B55] Stasch J. Mohr B. Neuhaus A. H. (2018). Disentangling the interaction of sex differences and hemispheric specialization for face processing - Evidence from ERPs. Biol. Psychol. 136, 144–150. doi: 10.1016/j.biopsycho.2018.06.003 29908225

[B56] Stekelenburg J. J. de Gelder B (2004). The neural correlates of perceiving human bodies: an ERP study on the body-inversion effect. NeuroReport 15, 777–780. doi: 10.1097/00001756-200404090-00007 15073513

[B57] Toga A. W. Thompson P. M. (2003). Mapping brain asymmetry. Nat. Rev. Neurosci. 4, 37–48. doi: 10.1038/nrn1009 12511860

[B58] Vingerhoets G. Verhelst H. Gerrits R. Badcock N. Bishop D. V. M. Carey D. . (2023). Laterality indices consensus initiative (LICI): a delphi expert survey report on recommendations to record, assess, and report asymmetry in human behavioural and brain research. Laterality 28, 122–191. doi: 10.1080/1357650X.2023.2199963 37211653

[B59] Voyer D. (1996). On the magnitude of laterality effects and sex differences in functional lateralities. Laterality 1, 51–83. doi: 10.1080/713754209 15513029

[B60] Wager T. D. Phan K. L. Liberzon I. Taylor S. F. (2003). Valence, gender, and lateralization of functional brain anatomy in emotion: a meta-analysis of findings from neuroimaging. NeuroImage 19, 513–531. doi: 10.1016/S1053-8119(03)00078-8 12880784

[B61] Westerhausen R. (2019). A primer on dichotic listening as a paradigm for the assessment of hemispheric asymmetry. Laterality 24, 740–771. doi: 10.1080/1357650X.2019.1598426 30922169

[B62] Willems R. M. van der Haegen L. Fisher S. E. Francks C. (2014). On the other hand: including left-handers in cognitive neuroscience and neurogenetics. Nat. Rev. Neurosci. 15, 193–201. doi: 10.1038/nrn3679 24518415

[B63] Wyczesany M. Capotosto P. Zappasodi F. Prete G. (2018). (2018). Hemispheric asymmetries and emotions: Evidence from effective connectivity. Neuropsychologia 121, 98–105. doi: 10.1016/j.neuropsychologia.2018.10.007 30352221

[B64] Zhang J. Zhou R. Oei T. P. S. (2011). The Effects of Valence and Arousal on Hemispheric Asymmetry of Emotion. J.Psychophysiol. 25, 95–103. doi: 10.1027/0269-8803/a000045

