# Supplementary material for: Hemispheric segregation is evident in early perception, not just higher cognition
Source: Front Psychol. 2026 Sep 17;17:1879495. doi: 10.3389/fpsyg.2026.1879495 (PMC13627375; doi:10.3389/fpsyg.2026.1879495)
Supplement: Supplementary file 1 [file Data_Sheet_1.docx]

Supplement A: Calculations for P1

# 1 Patterns of Hemispheric Asymmetry across Tasks

As the P1 is one of the earliest components associated with early cognition, we investigated lateralization and segregation patterns of the mean P1 amplitude exploratorily.

To assess if most participants show the either left- or rightward P1 lateralization, we investigated each of the tasks separately (see Supplementary Figure 1). It can be observed that across all tasks, right-handers show an overall right-lateralized P1, especially during the faces and emotions task. The binomial test however suggests, that only mixed-handed females during the emotions task (85.7% right-lateralized; p.adj=.027) show significantly more right- than leftward lateralization. No other group showed such a pattern during any of the tasks, suggesting that leftward and rightward lateralization do not differ within each task and group.

**Supplementary Figure 1.** Hemispheric functional lateralization of the mean P1 amplitude for left-, mixed-, and right-handed females (left) and males (right).

# 2 Patterns of Hemispheric Segregation

Across all participants different patterns of hemispheric segregation emerged. Supplementary Figure 2 shows the distribution across all participants. A pattern where all P1 amplitudes are larger over the right hemisphere is the most common pattern (39.8%), followed by a pattern where the P1 amplitude is larger over the right for faces and emotions, but larger over the left for words (23.5%). Importantly: this pattern of left-hemispheric lanuage and right-hemispheric emotions and faces is the expected typical pattern for the N1/N170 amplitudes, which was shown in 36.7% of participants. A pattern of all left P1 amplitudes is shown by 12.2% of participants, and 10.2% show a pattern where the P1 amplitude is larger over the left for faces and emotions, but larger over the right for words. All other patters occure with decreasing frequency (5.1% of words and faces being lateralized to the left and emotions to the right; 5.1% of words and emotions being lateralized to the left and faces to the right; 3.1% of language and faces being lateralized to the right and emotions to the left; 1.0% of language and emotions being lateralized to the right and faces to the left).

**Supplementary Figure 2.** Patterns of phenotyping regarding hemispheric segregation of the mean P1 component within the whole sample. “F” denotes the faces task, “L” the language task, and “E” the emotional faces task.

# 3 Hemispheric Segregation by Sex and Handedness

Investigating the influence of sex and handedness on the segregation of the P1 mean amplitude, shows that the “overrepresentation” of the “all right” patterns seem to differ between male right- and non-right-handers (see Supplementary Figure 3). While 50% of the male right-handers showed larger P1 amplitudes over the right hemisphere for all three tasks, only 20% of mixed- and 7.7% of left-handers showed such a pattern. For females this picture looks more evenly distributed, with 50% of right-handed, 52.4% of mixed-handed, and 35.7% of left-handed females showing the “all right” P1 pattern. Testing if right-, mixed-, and left-handed participants differ in their P1 segregation patterns, we conducted X^2^-test separately for males and females. Even though none of the X^2^-tests did reach significance (males: X^2^=16.59, p=.138; females: X^2^=14.67, p=.417), residual analysis for the male participants suggest a higher than expected proportion of “all left” P1 patterns (40.0%; RSM=2.42) for mixed-handed males, a higher than expected proportion of “all right” patterns (50%, RSM=2.68) for right-handed males, and a lower than expected “all right” pattern (7.7%, RSM=-2.16) in left-handed males.

**Supplementary Figure 3**. Percentages of all possible P1 segregation patterns across left-, mixed-, and right-handed females (left) and males (right).
