# Supplementary material for: Hemispheric segregation is evident in early perception, not just higher cognition
Source: Front Psychol. 2026 Sep 17;17:1879495. doi: 10.3389/fpsyg.2026.1879495 (PMC13627375; doi:10.3389/fpsyg.2026.1879495)
Supplement: Supplementary file 2 [file Data_Sheet_2.docx]

Supplement B: Robustness of choice of amplitude measure

In the main analysis, lateralization was determined from peak-to-peak amplitudes in the faces and emotional faces tasks and from mean amplitudes in the words task. To allow readers to assess the impact of this choice, we repeated the main parts of the analysis pipeline using the opposite measures: mean amplitude in the faces and emotional faces tasks and peak-to-peak amplitude in the words task. All other steps — electrode selection, hemispheric averaging, the direction-of-lateralization criterion, the aggregation of patterns (TFS, rTFS, 1rFS, 2rFS), and all binomial and chi-square tests — were identical to those in the main analysis. However, we limited the analyses to section 1 (hemispheric asymmetries across all tasks), section 2 (all patterns of hemispheric segregation), and section 7 (all patterns of hemispheric segregation by sex and handedness) to make this part of the supplements more concise.

# 1 Peak-to-Peak amplitude across all tasks

## 1.1 Patterns of Hemispheric Asymmetry across Tasks

As can be seen in Supplementary Figure 1, the patterns for the faces and emotional faces do not deviate from the main analysis – they are only repeated here to allow a direct comparison. However, the proportion of typical and atypical lateralization for the words task (using the peak-to-peak amplitude) did change. While right-handers showed 86.4% typical lateralization using the mean amplitude, only 50% showed a typical lateralization using the mean amplitude. Except for left-handed females (where 71.4% show typical lateralization), all other participant groups seem to show almost equal parts of typically and atypically lateralized participants. This is also reflected in the chi-square test, which did not show significant differences between the groups for the words task (Words: X^2^=3.28, p.adj=.706, V=.00, 95% CI [.00,.18]). None of the pairwise comparisons between typical and atypical lateralization during the words task got significant, for any of the participant groups (all p.adj > .999).

**Supplementary Figure 1.** Hemispheric functional lateralization of left-, mixed-, and right-handed females (left) and males (right) using the peak-to-peak measure across all tasks. For the emotional faces (top) and faces (middle) tasks the typical lateralization pattern is a rightward N1 lateralization, while for the words task (bottom) the typical lateralization is thought to be leftward.

## 1.2 Patterns of Hemispheric Segregation

The overall pattern of hemispheric segregation using the peak-to-peak measure for all tasks can be seen in Supplementary Figure 2. Comparing the segregation patterns from the main analysis with the patterns resulting from the “all peak-to-peak” analysis shows that both approaches differ from each other. While the typical segregation pattern was the most common in the main analysis, the analysis using the peak-to-peak amplitude shows the highest proportion of the pattern with reversed language (29.6% vs. 14.3% in the main analysis). This is followed by the pattern with reversed faces and emotional faces (22.4% vs. 26.5% in the main analysis). Typical segregation was only found in 21.4% (vs. 36.7% in the main analysis) of participants. The rTFS pattern was shown by 10.2% of participants (vs. 6.1% in the main analysis). A pattern of faces and language reversed was only shown by 1.0% of participants (vs. 2% in the main analysis). A pattern of reversed faces (2.0% in the main analysis), as well as a pattern of reversed emotional faces and language were found in 3.1% (this was also 3.1% in the main analysis) of participants, each.

**Supplementary Figure 2.** Patterns of phenotyping regarding hemispheric segregation using the peak-to-peak amplitude for all tasks within the whole sample. “F” denotes the faces task, “L” the language task, and “E” the emotional faces task.

## 1.3 Hemispheric Segregation by Handedness and Sex across all Patterns

As can be seen in Supplementary Figure 3, typical lateralization is reduced in each of the groups (compared with the main analysis). This seems to be mostly driven by the increased prevalence of reversed language functions across all groups.

**Supplementary Figure 3.** Percentages of all possible segregation patterns across left-, mixed-, and right-handed females (left) and males (right) using the peak-to-peak amplitude.

# 2 Mean amplitude across all tasks

## 2.1 Patterns of Hemispheric Asymmetry across Tasks

Investigating the hemispheric lateralization across all three tasks using the mean amplitude across all three tasks shows almost a reversal of typical and atypical lateralization for the faces and emotional faces task, compared to the main analysis. The corresponding percentages can be found in Supplementary Figure 4). While in the main analysis 63.6% of right-handed males showed a typical lateralization during the emotional faces task, the analysis using the mean amplitude suggests that only 31.8% show typical lateralization. The same holds true for the faces task, where 68.2% of right-handed males showed typical lateralization in the main analysis, while the analysis using the mean amplitude reveals only 32.8% of typical lateralization. The same can also be observed for right- and mixed-handed females. However, left- and mixed-handed males show very similar percentages when using the peak-to-peak (main) analysis or the mean amplitude (70% atypical lateralization the main analysis vs. 80% and 70% atypical lateralization in the emotional faces and faces task, respectively, using the mean amplitude).

**Supplementary Figure 4**. Hemispheric functional lateralization of left-, mixed-, and right-handed females (left) and males (right) using the mean amplitude. For the emotional faces (top) and faces (middle) tasks the typical lateralization pattern is a rightward N1 lateralization, while for the words task (bottom) the typical lateralization is thought to be leftward.

## 2.2 Patterns of Hemispheric Segregation

The patters of hemispheric segregation using the mean amplitude across all tasks can be found in f Supplementary Figure 5. Here the pattern with reversed faces and emotional faces shows the highest proportion with 51.0% of participants showing the pattern. This is followed by the typical pattern, which is only shown by 14.3% of participants (vs. 36.7% in the main analysis). A pattern of reversed language is found in 11.2% of participants (vs. 14.3% in the main analysis). The rTFS pattern was shown by 8.2% (vs. 6.1 in the main analysis). A reversal of emotional faces and language, as well as of only faces was shown by 5.1% of participants. The rarest pattern was a pattern of reversed face and language lateralization, with only 1.0% of participants showing such a pattern.

**Supplementary Figure 5.** Patterns of phenotyping regarding hemispheric segregation within the whole sample using the mean amplitude. “F” denotes the faces task, “L” the language task, and “E” the emotional faces task.

## 2.3 Hemispheric Segregation by Handedness and Sex across all Patterns

Supplementary Figure 6 shows the hemispheric segregation across all possible patterns, separate for male and female left-, mixed-, and right-handers. As reported before for the peak-to-peak amplitude, we observe a reduction of typical segregation across all participants group, when compared with the main analysis. For the mean analysis, the main driver of the reduction seems to be the increased prevalence of a reversed face and emotional face pattern.

**Supplementary Figure 6**. Percentages of all possible segregation patterns across left-, mixed-, and right-handed females (left) and males (right) using the mean amplitude.
